# Angiogenic Activity of Breast Cancer Patients’ Monocytes Reverted by Combined Use of Systems Modeling and Experimental Approaches

**DOI:** 10.1371/journal.pcbi.1004050

**Published:** 2015-03-13

**Authors:** Nicolas Guex, Isaac Crespo, Sylvian Bron, Assia Ifticene-Treboux, Eveline Faes-van’t Hull, Solange Kharoubi, Robin Liechti, Patricia Werffeli, Mark Ibberson, Francois Majo, Michäel Nicolas, Julien Laurent, Abhishek Garg, Khalil Zaman, Hans-Anton Lehr, Brian J. Stevenson, Curzio Rüegg, George Coukos, Jean-François Delaloye, Ioannis Xenarios, Marie-Agnès Doucey

**Affiliations:** 1 The Vital-IT, SIB (Swiss Institute of Bioinformatics), University of Lausanne, Lausanne, Switzerland; 2 Ludwig Center for Cancer Research, University of Lausanne, Lausanne, Switzerland; 3 Centre du Sein, CHUV (Centre Hospitalier Universitaire Vaudois), University of Lausanne, Lausanne, Switzerland; 4 Department of Oncology, CHUV (Centre Hospitalier Universitaire Vaudois), University of Lausanne, Lausanne, Switzerland; 5 Hopital Ophtalmique Jules-Gonin, Lausanne, Switzerland; 6 Merck Group, Darmstadt, Germany; 7 Harvard Medical School, Boston, Massachusetts, United States of America; 8 Institute of Pathology, University of Lausanne, Switzerland and Institute of Pathology, Johannes Gutenberg University, Mainz, Germany; 9 Department of Medicine, University of Fribourg, Fribourg, Switzerland; 10 Department of Gynecology and Obstetrics, CHUV (Centre Hospitalier Universitaire Vaudois), University of Lausanne, Lausanne, Switzerland; Johns Hopkins University, United States of America

## Abstract

Angiogenesis plays a key role in tumor growth and cancer progression. TIE-2-expressing monocytes (TEM) have been reported to critically account for tumor vascularization and growth in mouse tumor experimental models, but the molecular basis of their pro-angiogenic activity are largely unknown. Moreover, differences in the pro-angiogenic activity between blood circulating and tumor infiltrated TEM in human patients has not been established to date, hindering the identification of specific targets for therapeutic intervention. In this work, we investigated these differences and the phenotypic reversal of breast tumor pro-angiogenic TEM to a weak pro-angiogenic phenotype by combining Boolean modelling and experimental approaches. Firstly, we show that in breast cancer patients the pro-angiogenic activity of TEM increased drastically from blood to tumor, suggesting that the tumor microenvironment shapes the highly pro-angiogenic phenotype of TEM. Secondly, we predicted *in silico* all minimal perturbations transitioning the highly pro-angiogenic phenotype of tumor TEM to the weak pro-angiogenic phenotype of blood TEM and *vice versa. In silico* predicted perturbations were validated experimentally using patient TEM. In addition, gene expression profiling of TEM transitioned to a weak pro-angiogenic phenotype confirmed that TEM are plastic cells and can be reverted to immunological potent monocytes. Finally, the relapse-free survival analysis showed a statistically significant difference between patients with tumors with high and low expression values for genes encoding transitioning proteins detected *in silico* and validated on patient TEM. In conclusion, the inferred TEM regulatory network accurately captured experimental TEM behavior and highlighted crosstalk between specific angiogenic and inflammatory signaling pathways of outstanding importance to control their pro-angiogenic activity. Results showed the successful *in vitro* reversion of such an activity by perturbation of *in silico* predicted target genes in tumor derived TEM, and indicated that targeting tumor TEM plasticity may constitute a novel valid therapeutic strategy in breast cancer.

## Author Summary

Tumor vascularization is essential for tumor growth and cancer progression. In breast cancer, monocytes are angiogenic, i.e. able to induce tumor vascularization. In patients, blood circulating monocytes drastically increase their angiogenic activity when reaching the tumor, suggesting that the tumor microenvironment shapes their angiogenic activity. The identification of the tumor signals inducing the angiogenic activity of monocyte is of paramount significance because it represents the rationale for anti-angiogenic therapies in breast cancer. This goal was achieved by constructing an integrative model of monocyte behavior based on experimental data. The model predicted treatments abrogating the angiogenic activity of monocytes, which were experimentally validated in monocytes isolated from patient breast carcinoma. Importantly, these treatments reverted angiogenic monocytes into immunological potent cells. The main outcome of this modeling strategy for experimental and clinical oncology is the identification of effective treatments abrogating the angiogenic activity of monocytes and thus simultaneously revealing their functional plasticity.

## Introduction

Elucidating the various cell signaling cascades, pathway crosstalk, and how they influence final cell fate and behavior is crucial for defining therapeutic intervention points aimed at driving a cell towards a desired state. To this end, modeling approaches can be used to perturb a biological system *in silico* to test hypotheses on a scale that would be unfeasible to test experimentally. Boolean models have been extensively used in the past to simulate the behavior of cells based on their network activity [1]. In a Boolean modeling approach, the nodes in a regulatory network represent the state of activation of a gene (protein, receptor or ligand) using discrete variables (On or Off). The state of the network at a given instant can change depending on the state of the other nodes and can ultimately stabilize into attractors of either a single state (steady state) or an oscillating set of states (cycling attractors) [2]. Introducing perturbations in a biological regulatory network can change the attractors and even transition the system from one attractor to another one. The Boolean steady state of the network has been shown to correspond to the cellular states for various regulatory networks in the past [3]. Boolean modeling of steady state transitions helps in understanding the influence of perturbations on system wide behavior and has been used to identify the key molecular mechanisms controlling gene expression [4,5,6] and regulation [7,8], cell differentiation [9] and signal transduction [10,11,12,13,14,15,16,17,18,19,20]. Most of these models were developed in synergy by wet and dry laboratories. However, to date, only few of them have reported experimental validations (in primary cells) of the proposed *in silico* predictions [12,15,16]. In the present study we describe the application of a Boolean modeling based approach to investigate the molecular mechanisms underlying the angiogenic function of tumor monocytes from breast cancer patients and the experimental validation of *in silico* predictions derived from this modeling.

The formation of tumor-associated vasculature, a process also referred to as tumor angiogenesis, is essential for tumor progression. Tumor vessels can form from local pre-existing capillaries. This process is promoted by the recruitment of bone marrow-derived angiogenic cells (i.e. mainly monocytes, dendritic cells and neutrophils) at tumor sites [21,22,23]. Clinical studies have demonstrated in a variety of human solid tumors a positive correlation between increased micro-vessel density, infiltration of tumor-associated macrophages (TAM) [24] and unfavorable prognosis in cancer patients [25,26,27,28,29,30,31,32,33]. Recently, monocytes expressing the TIE-2/tek receptor tyrosine kinase (TEM: TIE-2 expressing monocytes) have been identified in peripheral blood and tumors of humans and mouse [34,35]. In experimental mouse models, TEM recruited to tumors accounted for apparently all angiogenic activity of bone marrow-derived cells since their selective ablation fully suppressed angiogenesis and induced tumor regression [34]. Hence, TEM appear to be key players in tumor angiogenesis but the tumor micro-environmental signals and the related signaling pathways governing their functions remain to be elucidated. Of particular interest from a disease standpoint is how TEM can be directed away from potentiating tumor angiogenesis and progression to monocytes being immunologically potent cells.

The VEGFR-1 (Vascular Endothelial Growth Factor Receptor-1), TGFBR-1 (Tumor Growth Factor β Receptor-1), TNF-R1 (Tumor Necrosis Factor Receptor-1) pathways have been reported to regulate tumor angiogenesis [36,37], but their activities have not been examined in human TEM. While we have previously reported that TIE-2 and VEGFR kinase activities drive immunosuppressive function of TEM in human breast Cancer [38], in this study, we investigated the contribution of these pathways along with TGFBR-1 and TNF-R1 pathways to TEM pro-angiogenic activity. We observed that the pro-angiogenic activity of TEM increased drastically from blood to tumor in breast cancer patients. We constructed an integrative and predictive model of TEM behavior to predict *in silico* all minimal perturbations that can transition the highly pro-angiogenic phenotype of breast tumor TEM into a weak pro-angiogenic phenotype and *vice versa*. By experimentally validating our computational predictions, we demonstrate here that the inferred regulatory network captured accurately patient TEM behavior. Thus, the contribution of the computational approach was not only essential to predict and tune TEM pro-angiogenic activity but also to identify the key underlying components and pathways of their pro-angiogenic activity. Finally, gene expression profiling of TEM transitioned to a weak pro-angiogenic phenotype confirmed that TEM infiltrating carcinoma of the breast remain plastic cells that can be reverted from pro-angiogenic and protumoral cells to immunological potent monocytes.

## Results

### TEM from peripheral blood and tumor tissue of breast cancer patients show distinct pro-angiogenic phenotypes

**Fig 1:**
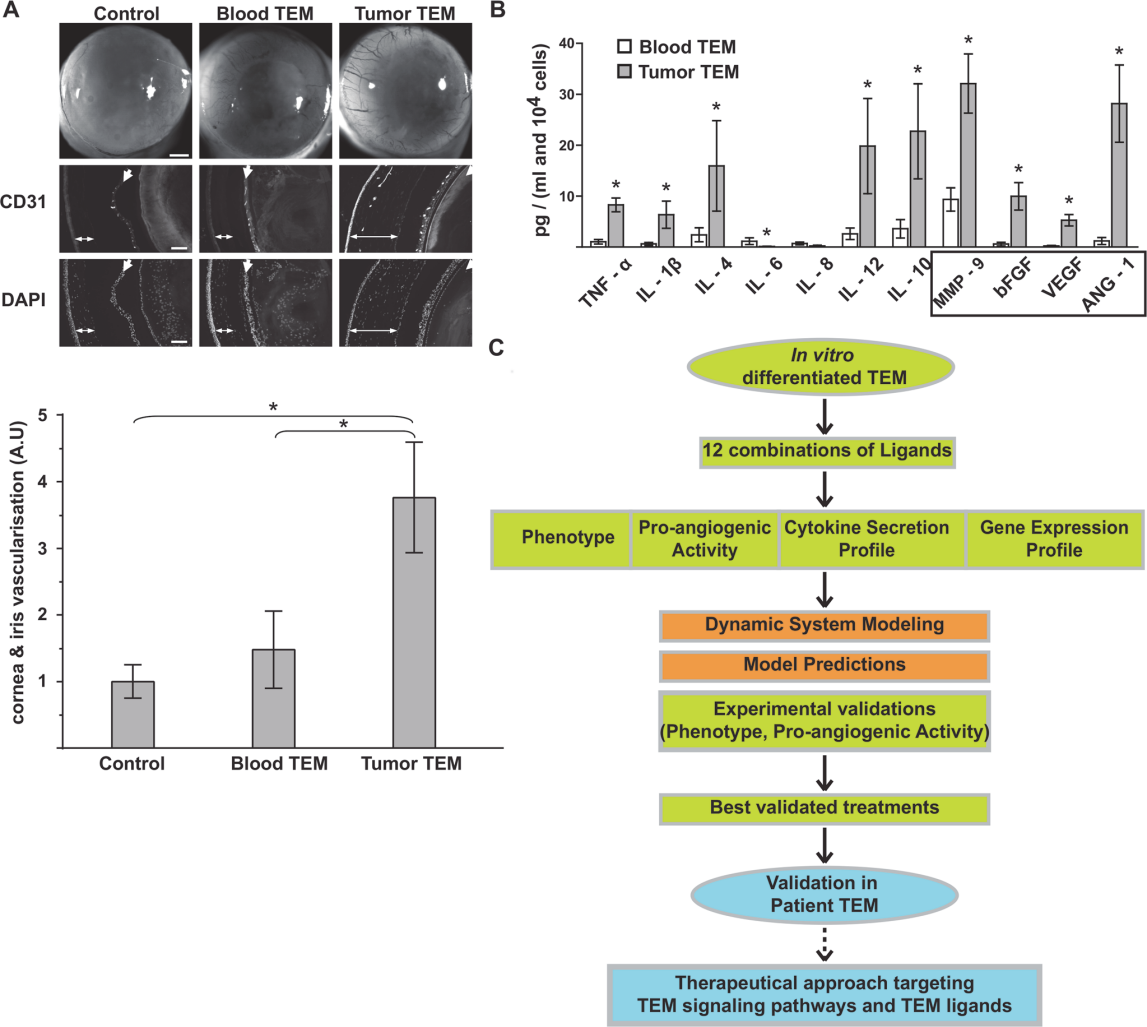
Phenotypical signature of pro-angiogenic TEM. (**A**) *In vivo* corneal vascularization assay to assess the pro-angiogenic activity of TEM isolated from peripheral blood and tumor of breast cancer patients. Bright field pictures of the eyes and fluorescent microscopy images of sagittal sections of the eyes stained with CD31 (stains specifically blood vessel endothelial cells) and Dapi (stains cell nucleus) are shown. Double-head and single arrows depict cornea and iris respectively. Corneas of control eyes were injected with buffer alone and show similar vascularization to uninjected eyes. Note the presence of blood vessels in the cornea injected with tumor TEM (double-head arrow) and its absence in the corneas injected with buffer (control) or blood TEM. Shown are representative data of 10 experiments. Bars in bright field and fluorescent images are 500 and 100 μm respectively. Bar graph represents a quantification of the vascular network of the cornea and the iris. (**B**) Secretion profile of cytokines and angiogenic factors in TEM isolated from patient blood and tumor. Angiogenic factors are boxed. Shown are cumulated data of 5 experiments, significant variations (P < 0.05) are indicated with an asterisk. (**C**) Workflow diagram of the strategy combining experimental and computational approaches to discover anti-angiogenic therapies. Green: experiments using ivdTEM. Blue, experiments using patient TEM; red: computational approach.

The angigoenic profile of TEM was investigated in a group of 40 newly diagnosed breast cancer patients (Table 1). We characterized by flow cytometry the phenotype of TEM from patient peripheral blood and freshly dissociated tumor specimens obtained at time of surgery (see Material and Methods). Based on our immunostaining and flow cytometry protocol we observed that TEM did not constitute a distinct subset of monocytes. In contrast, all monocytes showed expression of TIE-2, which was particularly low in patient blood and substantially higher on monocytes isolated from tumor tissue (S1 Fig. and Table 2). Thus, CD11b+, CD14+ monocytes from patient blood and tumor tissue were referred to as “TEM” and compared with respect to receptor and cytokine expression. However, tumor TEM co-expressed VEGFR-1 and TGFR-1 at significantly higher levels compared to peripheral blood TEM (Table 2). We next assess the pro-angiogenic activity of TEM using the *in vivo* corneal vascularization assay [39]. The cornea itself is avascular and was injected with TEM isolated from patient peripheral blood and tumor tissue. Thus, any growth of new vessels from the peripheral limbal vasculature must be due to injected TEM and reflect their pro-angiogenic activity. Tumor TEM showed a heterogeneous and consistently high pro-angiogenic activity inducing cornea and iris vascularization. By contrast, blood TEM were unable to induce *de novo* vascularization of the cornea but did increase the pre-existing vascular network of the iris (Fig. 1A). Thus, tumor and blood TEM show distinct pro-angiogenic phenotypes with the expression levels of TIE-2, VEGFR-1 and TGFR-1 mirroring their pro-angiogenic activity (Fig. 1A and Table 2). Finally, secretions were profiled in the conditioned medium of patient-isolated TEM and revealed that tumor TEM are paracrine inducers of tumor angiogenesis by releasing high levels of angiogenic factors (i.e. VEGF, bFGF, and ANG-1) and MMP9 (matrix metalloproteinase 9) (Fig. 1B). Blood and tumor TEM display a mixed M1-like (tumor-associated macrophages releasing inflammatory molecules) and M2-like (immunosuppressive macrophages polarized by anti-inflammatory molecules) phenotype, with secretion of both the pro- and anti-inflammatory cytokines IL-12 and IL-10, respectively (Fig. 1B). Given that TEM circulating in the blood infiltrate tumor tissue where they further differentiate [34], our data suggest that the tumor microenvironment shapes their highly pro-angiogenic phenotype.

**Table 1:** Clinical and pathological features of tumors and patients (n = 40).

Patient characteristics		%
Age, years		
	<50	29.6
	≥50	70.4
Surgical treatment		
	Mastectomy	32.1
	Tumorectomy	67.9
Lymph node status		
	Negative	74.5
	Positive	25.5
Tumor		
	T1	57.1
	T2< 3cm	42.9
Histology		
	Ductal	84.7
	Lobular	11.5
	Others	3.8
Grade		
	I	30.8
	II	38.4
	III	30.8

**Table 2:** Expression levels of receptors and integrin measured by flow cytometry at the surface of TEM.

	Tumor TEM	Blood TEM	ivdTEM
TIE-2	**4.09+/- 2.5**	**1.63+/- 0.71**	**2.56+/- 0.78**
VEGFR-1	**6.5+/- 3.24**	**1.09+/- 0.47**	**2.42 +/- 1.40**
TGFBR-1	**113.05+/- 29.1**	**2.08+/- 0.66**	**3.50+/- 1.13**
TNF-R1	1.39+/- 0.61	1.50+/- 0.76	1.69+/- 0.41
CCR5	1.03+/- 0.20	0.81+/- 0.17	1.03+/- 0.10
α5β1	1.94+/- 1.30	1.27+/- 0.18	nd
CXCR4	1.37+/- 0.4	2.18+/- 2.08	nd

Mean fold increase to isotype control antibodies +/- standard deviation (n = 10) is indicated. Significant differences (P<0.05) are in bold, nd not determined

### Combining computational and experimental approaches to delineate the pathways controlling TEM pro-angiogenic function

The identification of the ligands and the pathways controlling the highly pro-angiogenic activity of tumor TEM is of paramount significance because it represents the rationale for a treatment directing TEM away from being cells supporting tumor growth. The strategy we selected to reach this goal combined computational and experimental approaches to simulate and predict the behavior of patient TEM subjected to various ligand combinations. Given the limited amounts of patient specimens and the low frequency of TEM (TEM represented 6.7% ±2.5% of peripheral blood mononuclear cells and 22% ±2.7% of the tumor hematopoietic infiltrate), only a limited number of ligand combinations could be investigated experimentally. The availability of limited amounts of patient TEM was partially overcome by taking advantage of our recently developed model system of TEM differentiated *in vitro* by exposing CD34^+^ cord blood hematopoietic progenitors to breast cancer cell conditioned culture medium [38,40]. *In vitro* differentiated TEM (thereafter named ivdTEM) are angiogenic [38,40] and display an intermediate phenotype relative to blood and tumor TEM (Table 2). Consistent with their phenotype (Table 2), ivdTEM released intermediate amounts of angiogenic factors relative to blood and tumor TEM (Table 3).

**Table 3:** TEM release of angiogenic factors measured by flow cytometry.

	Tumor TEM	Blood TEM	ivdTEM
MMP9	**32.1+/- 5.7**	**9.36+/- 2.2**	**14+/- 3.1**
VEGF	**5.2+/- 2.1**	**0.21+/- 0.1**	**1.57 +/- 0.6**
ANG-1	**28.1+/- 7.5**	**1.2+/- 0.7**	**7.8+/- 1.3**

Mean secretions +/- standard deviation (n = 5) in pg/ml and 10^4^ cells are indicated. Significant differences (P<0.05) are in bold.

Moreover, the *in silico* modeling and predictions helped us to focus on the most clinically relevant monocytic ligands and to spare precious patient specimen. The workflow of our approach consists of five steps (Fig. 1C): 1) experimental measurement of the responses of TEM differentiated *in vitro* to a set of ligands, 2) construction of a dynamic regulatory network based on these experimental data, 3) *in silico* prediction of the treatments altering TEM behavior, 4) experimental validation of computationally predicted treatments using ivdTEM and 5) validation the best predicted treatments in patient TEM (Fig. 1C). Finally, to help shed light on possible molecular mechanisms underlying TEM pro-angiogenic transformation, we selected several treatment combinations and measured genome wide expression profiles for the TEM differentiated *in vitro*, comparing the state of the cells before and after treatment.

### Identification of critical ligands impacting the phenotype and pro-angiogenic activity of TEM differentiated *in vitro*—Antagonistic effect of TGF-β and synergistic effects of TNF-α on TEM pro-angiogenic phenotype and function

**Fig 2:**
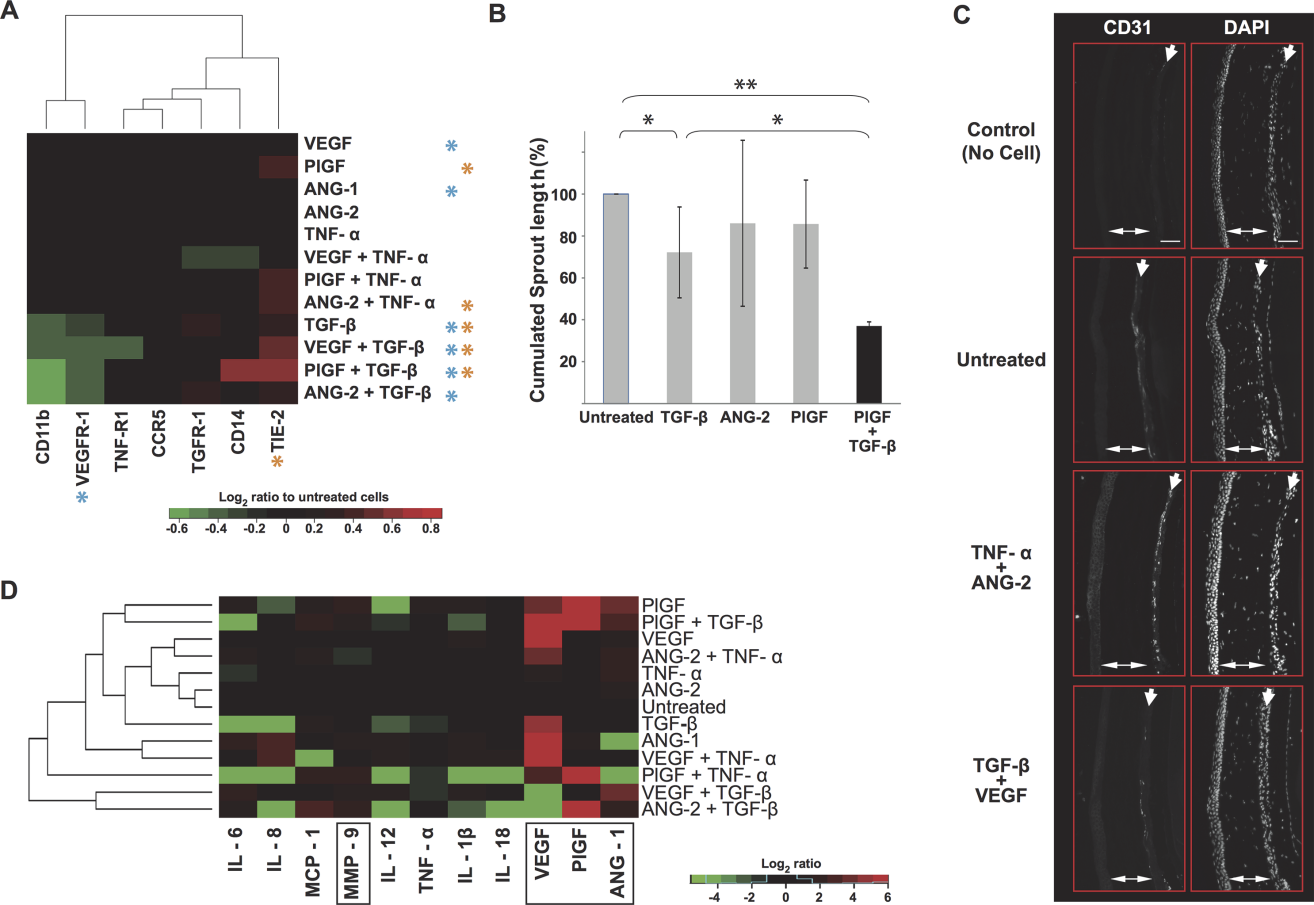
Synergistic and antagonistic effects of TNF-α, PlGF, ANG-2 and TGF-β on TEM pro-angiogenic phenotype. IvdTEM were exposed to different combinations of ligands and changes in the expression of receptors at their surface of was measured by flow cytometry 36 hours post-treatment and displayed as mean log_2_ ratios relative to untreated cells (**A**). Significant variations (P < 0.05, T test) in VEGFR-1 and TIE-2 expression in TEM are indicated with an asterisk in the heatmap. Changes in TEM pro-angiogenic activity in response to treatments was measures *in vitro* (**B**) and *in vivo* (**C**) using HUVEC sprouting assay and corneal vascularization assay respectively, 3 to 5 independent angiogenic assays were performed per condition. * P < 0.05, ** P < 0.01. (**D**) The secretion of cytokines and angiogenic factors in response to treatments was experimentally measured in the conditioned medium of the culture 36 hours post-treatments. The secretions of ivdTEM were mathematically inferred and displayed as mean log_2_ ratios relative to untreated cells. Angiogenic factors are boxed. Shown are cumulated data of 3 to 10 independent experiments (panels A and D), the corresponding experimental data and all P values are available in S2 and S3 Tables.

Our strategy was to expose TEM to several treatments to identify the ligands and pathways critically controlling their pro-angiogenic activity. TEM differentiated *in vitro* were exposed to angiogenic factors (VEGF, PlGF and ANG-1, ANG-2 which are the ligands of VEGFR-1 and TIE-2 respectively) in combination with either TGF-β or TNF-α and the changes in their phenotype, angiogenic activity and paracrine secretion profile were examined. These experimental results were used as the foundations for a computational model that would allow predicting treatments increasing or dampening TEM proangiogenic activity. First, changes in TEM phenotype were evaluated by flow cytometry 36h post treatment. Globally, treatments combined with TGF-β or TNF-α displayed a stronger impact on TEM phenotype than single treatments with however, the exception of TGF-β. Overall, CD11b, CD14, VEGFR-1 and TIE-2 expression displayed larger changes in response to treatment than CCR5, TNF-R1 and TGFBR-1 (Fig. 2A). A hallmark of TGF-β treatments was a strong decrease in VEGFR-1 and CD11b expression and an increase in TIE-2 expression (Fig. 2A). By contrast, TNF-α treatments had no impact on VEGFR-1 expression and TNF-α increased TIE-2 expression when combined with PlGF or ANG-2 (Fig. 2A).

We assessed the impact of the treatments on the pro-angiogenic activity of TEM using *in vitro* HUVEC (Human Umbilical Vascular Endothelial Cells) sprouting assay (see Methods). Treated TEM were applied to HUVEC grown on microcarrier beads and embedded in a fibrin gel to measure their aptitude to induce HUVEC sprouting i.e. the initial step of blood vessel formation. Single treatments show no significant impact on TEM proangiogenic activity relative to untreated cells with the exception of TGF-β which significantly reduced TEM pro-angiogenic activity (Fig. 2B). Interestingly, combining TGF-β with PlGF further decreased VEGFR-1 expression (Fig. 2A) and TEM proangiogenic activity (Fig. 2B) suggesting that TGF-β synergized with PlGF to reduce TEM proangiogenic activity. We examined the impact of combined treatments on TEM using *in vivo* corneal vascularization assay. Indeed, *in vitro* sprouting assay was preferred for quantification but is however less reliable because it does not recapitulate the intricate balance of signals from growth factors, mural cells and extracellular matrix of *in vivo* angiogenesis. TNF-α in combination with ANG-2 (or PlGF) significantly increased TIE-2 expression whilst leaving VEGFR-1 expression unchanged (Fig. 2A), and raised TEM pro-angiogenic activity (Fig. 2C. Cornea and iris vascularization in AU: control: 1; untreated: 1.81; TNF-α+Ang-2: 4.58). Conversely, TGF-β in combination with VEGF resulted in a comparable induction of TIE-2 but decreased VEGFR-1 expression (Fig. 2A), and reduced TEM pro-angiogenic activity (Fig. 2C. Cornea and iris vascularization in AU: TGF-β+VEGF: 1.36). Taken together these results show, for the first time, that both Tie2 and VEGFR1 pathways control TEM pro-angiogenic activity. Furthermore, TIE-2 and VEGFR1 pathways synergized with the TNF and TGF pathway to induce and reduce TEM pro-angiogenic activity respectively.

Finally, we examined the impact of the different ligand treatments on TEM secretions. Thus, cumulated TEM secretions from ivdTEM were measured experimentally and the secretions for TEM were mathematically inferred (ivdTEM correspond to double positive DP cell population, see Materials and Methods and S2 Fig.) and display in Fig. 2D. Of note, none of the single or double treatments we have examined experimentally (Fig. 2D) shifted completely the paracrine secretion profile of TEM differentiated *in vitro* toward that of blood or tumor TEM (compare Fig. 2D and 1B). These results suggest that transitioning ivdTEM into blood or tumor TEM requires a model to simulate computationally the impact of a larger number of ligand combinations on TEM behavior.

### Construction of dynamical models from the experimental data using TEM differentiated *in vitro*

**Fig 3:**
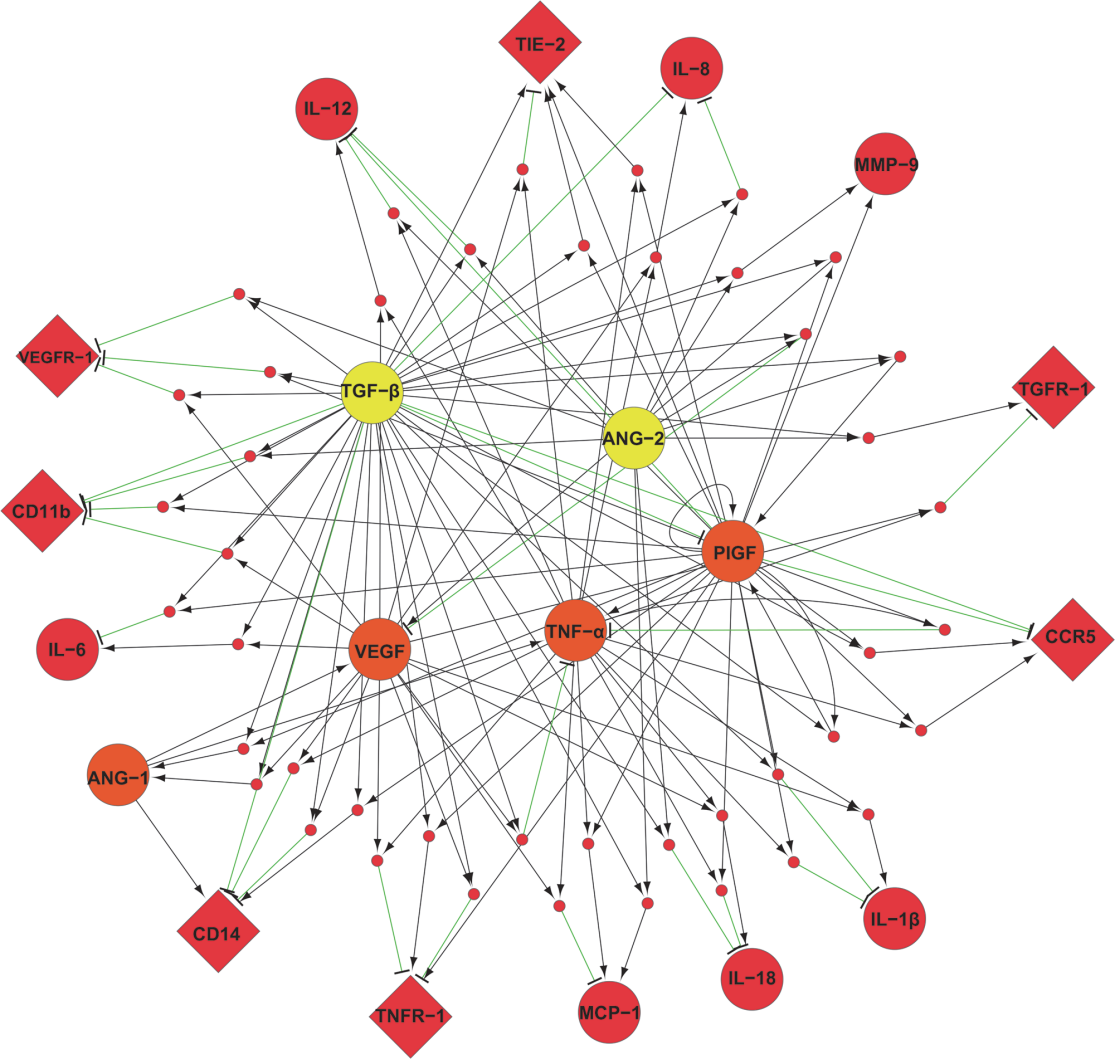
ivdTEM network topology. Dynamical models of treatments/receptors/cytokines interactions in ivdTEM. Inputs (treatments) and output (receptor and secreted soluble factors) are depicted in yellow and red respectively. Factors that were used as treatment and measured as output are depicted in orange. Combined treatments (network nodes i.e. AND) are represented as small pink circles. Stimulatory and inhibitory effects of single or combined treatments are depicted by black arrow-headed edges and green edges respectively. Circles and diamonds represent soluble factors and receptors respectively. All the links presented are provided in S4 Table. Boolean equations used for representing ivdTEM regulatory networks are provided in S6 Table.

The limited amounts of patient TEM and the combinatorial nature of the ligands precluded experimental testing of all the ligand combinations and was the rationale for building an integrative and predictive model of TEM behavior. We used TEM differentiated *in vitro* to derive a dynamical regulatory network from experimental data obtained with a selected number of ligands (Fig. 2) and used then as a proxy to assess the clinically most relevant ligand combinations. To create the models, data sets of receptor expression (Fig. 2 and S2 Table) and paracrine secretion profiles (Fig. 2 and S3 Table) were combined to infer relevant relationships (or links) between ligands and receptors. Briefly, relevant links were identified based on the amplitude of their expression or secretion changes, their reproducibility, and their coherent variations across the treatments (see Methods). Based on these criteria, amongst 924 possible links (7 receptors × 11 secreted factors × 12 treatments) we retained 74 relevant links (S4 Table). Globally, TNF-α, TGF-β and PlGF appeared as key regulators of TEM network. However, TNF-α in contrast to TGF-β, was strongly regulated by other factors (Fig. 3). Dynamical Boolean modeling was then performed by integrating the retained links into an algorithm for computing Minimal Intervention Set (MIS) of TEM regulatory network. Given a regulatory network, MIS patterns represent a set of simultaneous perturbations (or treatments) to force the network into a desired steady state, where a subset of nodes remain at a fixed expression level of either low or high [41,42]. The term minimal implies that no other sub-set of an MIS pattern can lead to the desired steady state behavior. However, for a given network, there can be more than one MIS patterns to generate the same steady state. The MIS algorithm proposed by Garg *et al* [43,44] was used for assessing TEM regulatory network by computationally predicting all possible set of up to three simultaneous treatments that can force the TEM network into a weakly (i.e. blood TEM) or highly (i.e. tumor TEM) pro-angiogenic phenotype.

### The plasticity of TEM predicted computationally was validated experimentally using TEM differentiated *in vitro*

Relative to their blood counterparts, tumor TEM display a higher pro-angiogenic activity (Fig. 1A), a paracrine profile shifted toward angiogenesis (Fig. 1B) and higher levels of TIE-2 and VEGFR-1 (Table 2). Therefore blood and tumor TEM can be viewed as two distinct cell steady state behaviors and ivdTEM as an intermediate state (Tables 2 and 3). Using the regulatory network model of TEM differentiated *in vitro* we predicted the minimal treatments required for transitioning tumor TEM to blood TEM and *vice versa*. Because the expression levels of TIE-2 and VEGFR-1 controlled (Fig. 2) and mirrored (Fig. 1B) TEM pro-angiogenic activity, we assigned to TIE-2 and VEGFR-1 nodes a fixed polarity of either both over-expressed or down-modulated for highly pro-angiogenic (i.e. tumor TEM) or weakly pro-angiogenic (i.e. blood TEM) steady states respectively. Computationally predicted minimal perturbations sets (MIS) are reported in Table 4. It is interesting to note that all the predicted treatments were composed of at least two, and mostly three simultaneous perturbations. Only one treatment, combining three perturbations, was predicted by the model to promote TEM pro-angiogenic activity (TNF-α, ANG-2 and PlGF; Table 4). Conversely, eleven distinct treatments were predicted to dampen TEM proangiogenic activity and resulted in three main groups (Table 4). The first group of treatments combined TIE-2 tyrosine kinase inhibitor with TGF-β and a ligand of VEGFR-1 or TIE-2. Treatments from the second group involved VEGFR-1 kinase inhibitor, and the third group of treatments associated TGF-β with TNF-α and a ligand of TIE-2 or VEGFR-1 (Table 4). It is worth noting here that we assumed that possible compensatory mechanisms resulting from the blocking of the receptor signaling (rather than knocking down the receptor) do not significantly affect the angiogenic activity. Results showed that this assumption was valid for the particular case of the receptors under study.

**Table 4:** Computationally predicted minimal perturbations sets (MIS) required for transitioning TEM regulatory network into highly or weakly pro-angiogenic TEM.

		RTK activity inhibited	Up-regulated Inflammatory Ligand	Up-regulated VEGFR-1 ligand	Up-regulated TIE-2 ligand
**Transition to weakly pro-angiogenic TEM**					
	**Group 1**				
		TIE-2	TGF-β	VEGF	-
		TIE-2	TGF-β	PlGF	-
		TIE-2	TGF-β	-	ANG-1
		TIE-2	TGF-β	-	ANG-2
	**Group 2**				
		VEGFR-1	TNF-α	-	ANG-1
		VEGFR-1	TNF-α	VEGF	-
		VEGFR-1	-	all but PlGF	-
		TIE-2 and VEGFR-1	-	-	-
	**Group 3**				
		-	TNF-α and TGF-β	PlGF	-
		-	TNF-α and TGF-β	-	ANG-1
		-	TNF-α and TGF-β	VEGF	-
**Transition to highly pro-angiogenic TEM**		-	TNF-α	PlGF	ANG-2

These two final desired cell steady states were obtained by assigning to TIE-2 and VEGFR-1 nodes a fixed polarity of either both high (highly pro-angiogenic i.e tumor TEM) or low (weakly proangiogenic i.e. blood TEM) expression levels. Computationally predicted MIS decreasing TEM pro-angiogenic activity were classified in three groups based on the receptor tyrosine kinase (RTK) inhibited and inflammatory (TGF-β or TNF-α) and angiogenic ligands up-regulated.

With currently available tools, VEGFR-1 kinase activity is almost impossible to manipulate. Indeed, to date, all available VEGFR-1 kinase inhibitors also inhibit VEGFR2 and VEGFR3 to a lesser extent, thus preventing experimental validation of any treatment of the second group. For experimental validations, we therefore selected the combined TNF-α/ANG-2/PlGF treatment, predicted to promote angiogenesis, two treatments of the first group (TIE-2inhibitor/TGF-β/PlGF, and TIE-2 inhibitor/TGF-β/ANG-2), one treatment of the third group (PlGF/TGF-β/TNF-α) and a TIE-2 kinase inhibitor alone. These experimental validations were first conducted in TEM differentiated *in vitro*.

**Fig 4:**
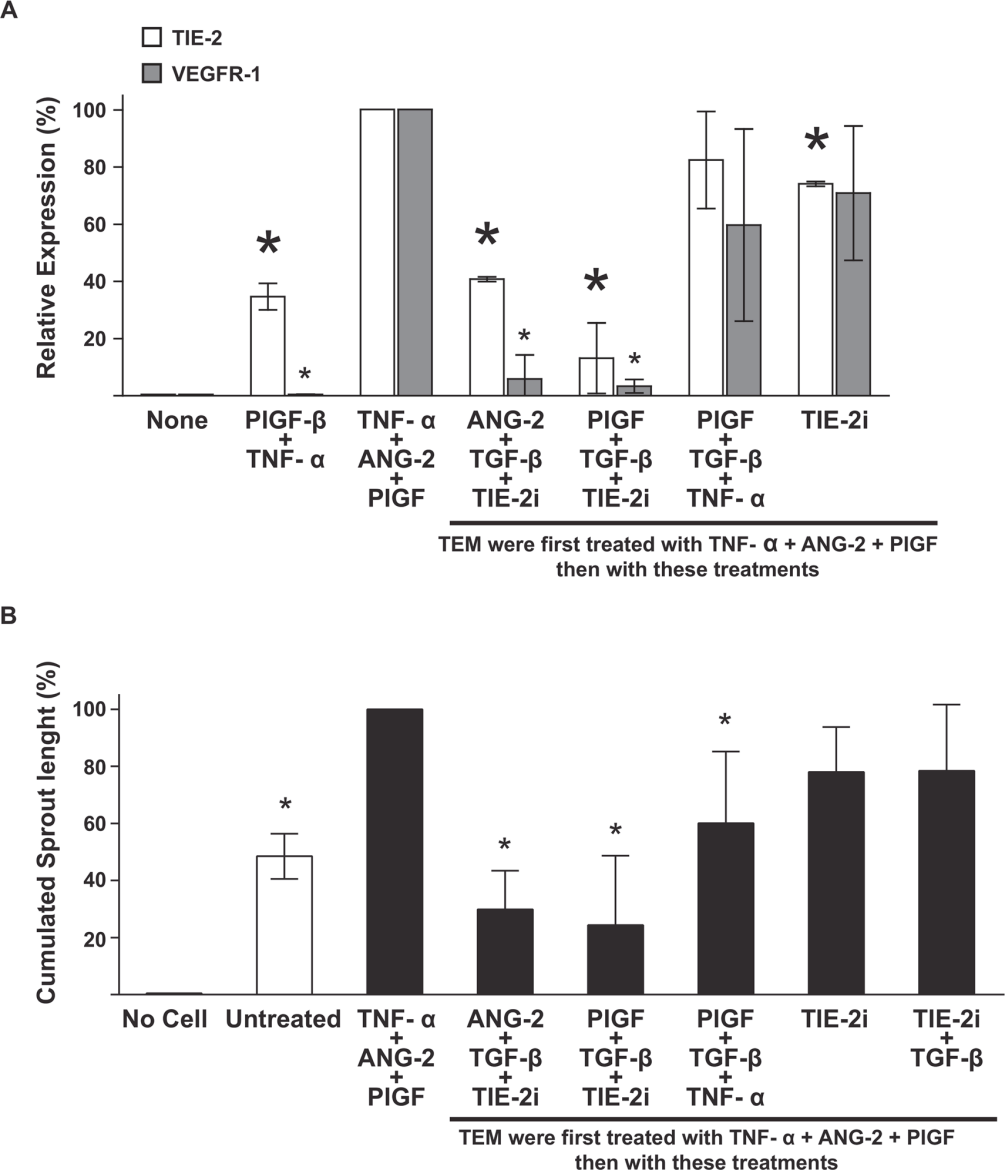
Experimental validation of *in silico* predicted treatments using ivdTEM. TEM differentiated *in vitro* were exposed to the treatments predicted *in silico* and their changes in TIE-2 and VEGFR-1 expression (**A**) and pro-angiogenic activity (**B**) measured by flow cytometry and *in vitro* angiogenesis sprouting assay respectively. The impact of inhibitory treatments was examined on ivdTEM previously treated with TNF-α/PlGF/ANG-2 (panels A and B). Significant variations (P < 0.05) are indicated with an asterisk. In panel A, small and large asterisks referred to VEGFR-1 and TIE-2 expression respectively.

As predicted, the TNF-α/ANG-2/PlGF combined treatment induced TIE-2 and VEGFR-1 expression (Fig. 4A) and increased their proangiogenic activity (Fig. 4B). Importantly, this combined treatment induced TIE-2 and VEGFR-1 expression and TEM pro-angiogenic activity more efficiently than PlGF/TNF-α (Fig. 4A) and PlGF or ANG-2 single treatments (Fig. 2A and B). These results validate our *in silico* prediction and reveal the synergistic effect of TIE-2, VEGFR-1 and TNF-α pathways in controlling TEM pro-angiogenic activity.

The predicted inhibitory effect of the other treatments was assessed on TEM pre-treated with TNF-α/ANG-2/PlGF, which display an increased pro-angiogenic phenotype compared to untreated cells (Fig. 4A and B). This pre-treatment increased the dynamic range and therefore the sensitivity of detecting inhibitory effects. TIE-2 kinase inhibitor/TGF-β combined with ANG-2 or PlGF significantly decreased TIE-2 and VEGFR-1 receptor expression (Fig. 4A) consistently reduced their pro-angiogenic activity (Fig. 4B). The PlGF/TGF-β/TNF-α treatment was not as effective, but still reduced their pro-angiogenic activity. These combined treatments were synergistic and minimal since TIE-2 kinase inhibitor (Fig. 4A and B) or single treatments alone (Fig. 2A and 2B) or double treatments (PLGF/TGF-β or TIE-2inhibitor/TGF-β display on Fig. 2B and Fig. 4B, respectively) were not sufficient to decrease VEGFR-1 expression and TEM pro-angiogenic activity. By contrast, PlGF/TGF-β/TNF-α heterogeneously decreased TIE-2 and VEGFR-1 expression (Fig. 4A) and TEM pro-angiogenic activity (Fig. 4B). In summary, from these validation experiments we found that the best computationally predicted treatment promoting TEM pro-angiogenic activity was TNF-α/ANG-2/PlGF and the best dampening activity was found using TIE-2 kinase inhibitor/TGF-β associated with a ligand of TIE-2 or VEGFR-1.

### ANG-2/ TGF-β and PlGF/ TGF-β treatments increased transcript abundance of genes regulating differentiation and immune response of TEM differentiated *in vitro*

**Fig 5:**
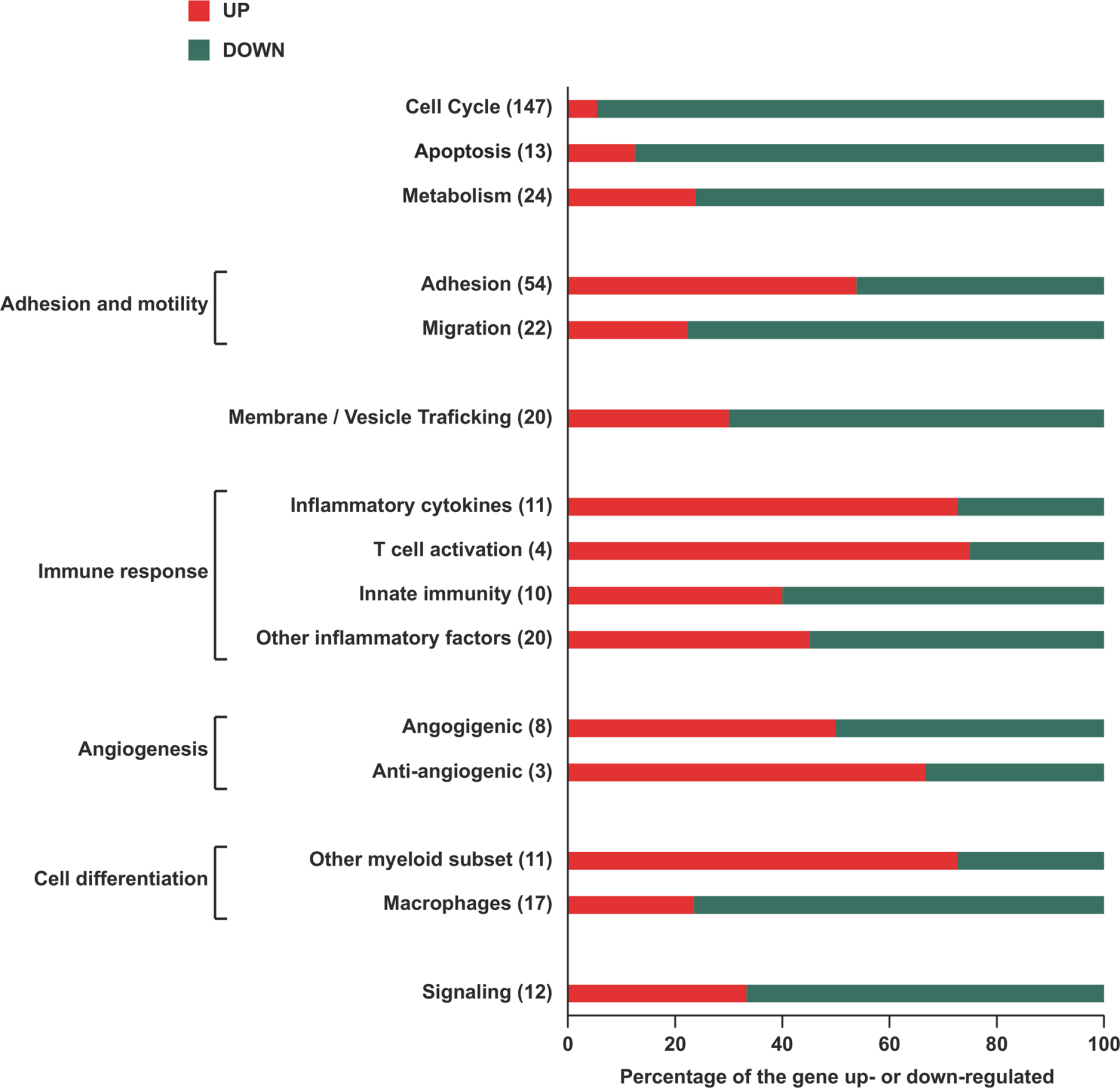
Gene expression changes in monocytes treated with TGF-β/ANG-2 and TGF-β/PlGF relative to untreated and VEGF/TNF-α cells. 398 significantly (P ≤ 0.05) differentially expressed genes were manually annotated and classified in categories (S5 Table). In each category the percentage of up- and down-regulated genes are displayed as well as the total number of genes (under brackets). 50 genes could not be assigned to these categories.

Having identified the critical ligands and pathways controlling TEM plasticity, we next examined in TEM differentiated *in vitro* whether differential gene expression might also contribute to the molecular basis of TEM plastic behavior. This analysis may shed light on the molecular mechanisms underlying the observed TEM responses. To this end, we selected VEGF/TNF-α, ANG-2/TGF-β and PlGF/TGF-β treatments for gene expression profiling using Affimetrix whole genome microarrays, because these treatments were present in 17, 16 and 14, respectively of the 74 links (treatment/receptor/cytokine) retained in TEM regulatory network (S4 Table and Fig. 3). All the other treatments occurred less frequently. Hierarchical clustering demonstrated that TGF-β-based treatments (ANG-2/TGF-β and PlGF/TGF-β) clustered separately from VEGF/TNF-α and control treatments. A total of 398 genes were significantly (p<0.05) and differentially expressed between the two clusters among which 369 and 72 genes were altered by TGF-β/ANG-2 and TGF-β/PlGF treatments respectively (S5 Table, NT unique lists) while 43 were regulated in common (S5 Table, NT intersect list). Enrichment analyses of the gene expression data against known pathways and functional gene categories were conducted as described in Materials and Methods. No enrichment of specific pathways of interest was observed due to the fact that the gene annotations were too general and did not correspond to specific functions of monocytes. Therefore, the 398 differentially expressed genes were annotated and classified in categories manually (S5 Table). Similar expression profiles were obtained for untreated and TNF-α/VEGF treated cells consistent with their weak impact on TEM functional angiogenic phenotype (Fig. 2). By contrast, ANG-2/TGF-β and PlGF/TGF-β treatments inhibited TEM pro-angiogenic activity (Fig. 2) and down-modulated the expression of pro-angiogenic genes (Fig. 5 and S5 Table). Furthermore and interestingly, the expression of VASH1 (vasohibin 1) and UCN (urocortin) genes coding for anti-angiogenic proteins was simultaneously up-regulated (Fig. 5 and S5 Table). In response to both ANG-2/TGF-β and PlGF/TGF-β treatments, 95% of the genes functionally related to the cell cycle displayed a down-modulated expression indicating that TEM stopped proliferating with profound changes in their metabolism but without, however undergoing apoptosis (the expression of metabolism and apoptosis related genes was down-modulated for 76% and 88% of them respectively). TEM treated with TGF-β/ANG-2 or TGF-β/PlGF show the expression of some genes (P2RY12, TMCC3, NPDC1, IFFO1, UBASH3B, C11orf52, SLC4A7, TMEM87A, NPL, EMB, PCNA, DNA2, TMEM86A, MMP12, CTSD, AXL, RASGRP3, TUBB, FCGR1A, CR1, MX2) previously ascribed to mouse TAM [45,46,47]. However, in response to ANG-2/TGF-β and PlGF/TGF-β treatments, TEM down-modulated the expression of genes involved in macrophage differentiation (Fig. 5) and started to acquire the profile (RGS1, CXCL11, CXCL9, STAT1, IFIH1, ISG20, NT5C3, ADC, PDGFRL, TNF-ASF12, IFIT5, RGS10, TRAF3IP3, CIDEB, APOBEC3A, PYGL, RRM1, MAF, NLRC4, IL10, MYC, DUT, POLE4, CXCL17) of dendritic cells matured *in vitro* by exposure to lipopolysaccharide and interferon-gamma [48,49]. Along these lines, genes encoding for dendritic cell markers, antigen processing and adaptive immune response were upregulated while genes involved in immune suppression show markedly decreased expression (Fig. 5 and [38]). Finally the expression of genes related to adhesion and migration were up- and down-regulated respectively indicating that TEM mobility was strongly reduced; an observation consistent with the arrest of their cell cycle and the alteration of their differentiation program (Fig. 5 and S5 Table). Along these lines, we observed experimentally that ivdTEM treated with PlGF/TGF-β/TIE-2i display reduced mobility towards the human epithelial tumor cell line MDA-231 (S3A Fig.) and slowed down the growth of MDA-231 cells (S3B Fig.)

Taken together, our results suggest that ANG-2/TGF-β and PlGF/TGF-β treatments are not only anti-angiogenic but also shift the gene expression profile of monocytes toward the one of cells promoting immune surveillance, thereby limiting tumor growth.

### Validation of the predictions in patient TEM- TGF-β/TIE-2 pathways may represent a therapeutic target to inhibit tumor TEM proangiogenic function

**Fig 6:**
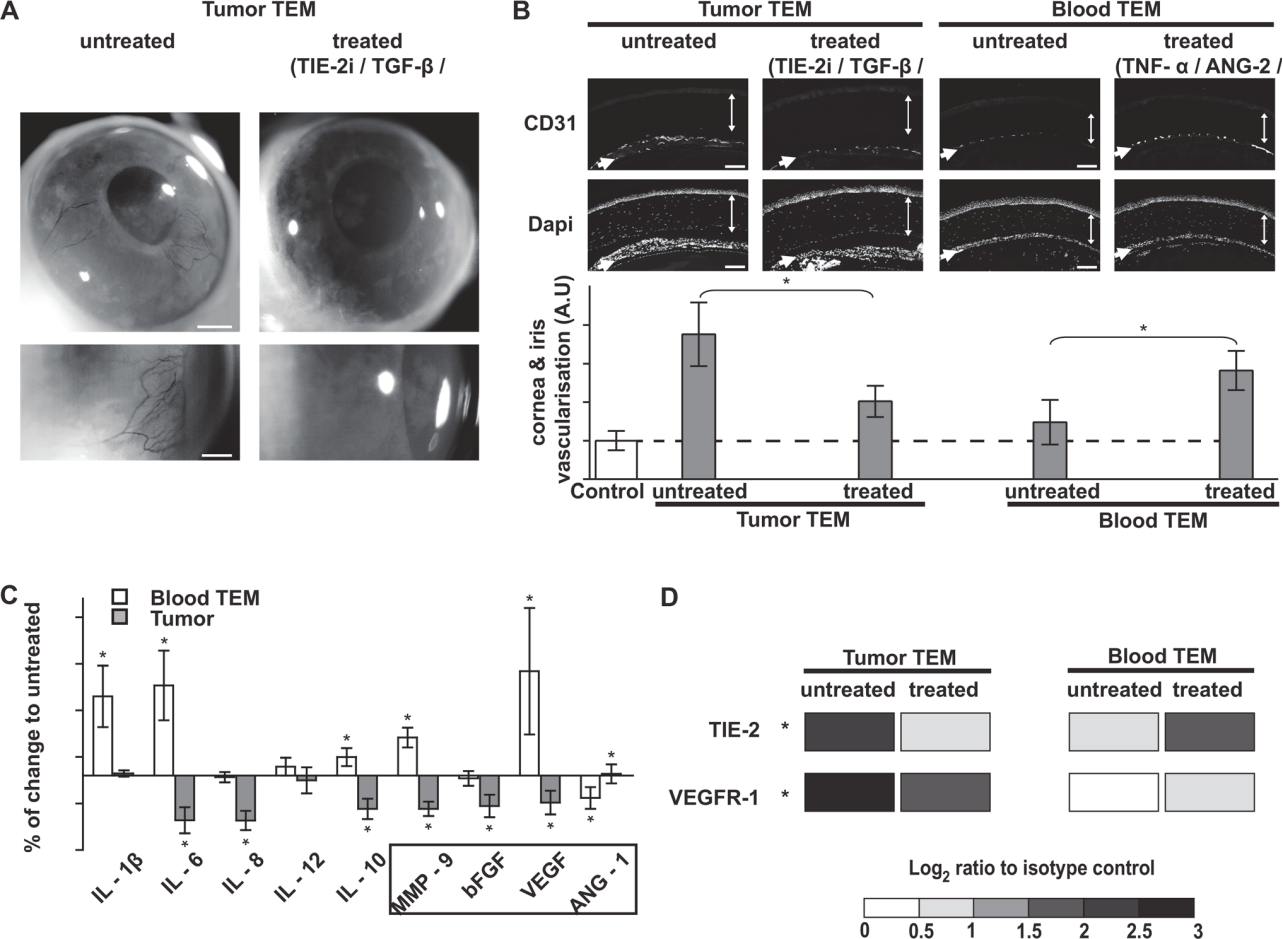
Controlling the pro-angiogenic activity of TEM from breast cancer patients. **(A and B)** *In vivo* corneal vascularization assay, as described in Fig. 1A, showing the variations in the pro-angiogenic activity of patient TEM in response to *in silico* predicted treatments. TIE-2 kinase inhibitor/TGF-β/VEGF treatment decreased tumor TEM pro-angiogenic activity while TNF-α/PlGF/ANG-2 treatment increased the pro-angiogenic activity of blood TEM. Bars are 500 and 250 μm in A and B respectively. Cornea and iris are depicted by double-head and single arrows respectively. Bar graph represents a quantification of the vascular network of the cornea and the iris. (**C**) Variations of patient blood and tumor TEM secretion profiles in response to TNF-α/PLGF/ANG-2 and TIE-2 kinase inhibitor/TGF-β/VEGF treatments. Angiogenic factors are boxed. No significant variations were detected for IL-4 and TNF-α. (**D**) Variations of patient blood and tumor TEM expression of TIE-2 and VEGFR-1 in response to the same treatments. Fold increase to isotype control antibody is indicated, similarly as in Table 2. Shown are cumulated data (C, D) or representative results (A and B) of 3 to 5 experiments. Significant variations (* P < 0.05, T test).

We next sought to validate the computationally predicted treatments in TEM isolated from patient breast carcinoma. Tumor TEM were exposed to TIE-2 kinase inhibitor combined with TGF-β and simultaneously engaged their VEGFR-1 using VEGF (alternatively PlGF, Table 4 and Fig. 4). This combined treatment strongly reduced the pro-angiogenic activity of tumor TEM in the mouse cornea vascularization assay (Fig. 6A and B) and decreased the expression of TIE-2 and VEGFR-1 (Fig. 6D). Furthermore, this treatment reduced the secretion of IL-6, IL-8, MMP9, bFGF and VEGF, consistent with a paracrine profile shifted toward a M1-like phenotype and closer to the one of blood TEM (Fig. 6C and 1B). Conversely, TEM from patient blood exposed to the combined treatment of TNF-α/PlGF/ANG-2 increased their pro-angiogenic activity in the mouse cornea vascularization assay (Fig. 6B) and was associated with significantly higher secretion of IL-1β, IL-6, IL-10, MMP9 and VEGF (Fig. 6C) and increased expression of TIE-2 and VEGFR1 (Fig. 6D). These results highlighted the validity of our combined experimental and computational approach to revert the pro-angiogenic phenotype of TEM and revealed, for the first time, that tumor TEM remain plastic cells representing attractive targets for anti-angiogenic therapies.

### ANG-2 and PIGF survival analysis on breast cancer patients

We addressed the question whether or not the expression levels of ANG-2 and PIGF when considering the overall breast tumor have impact on the survival (considered here as relapse free survival).

**Fig 7:**
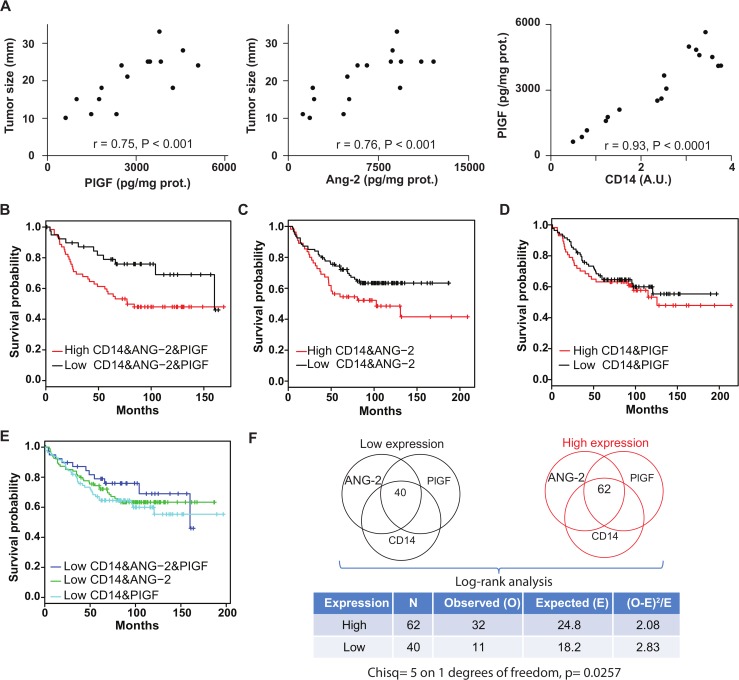
Tie-2 and VEGFR-1, and Ang-2 and PIGF represent attractive targets in breast cancer (A) Correlation of Ang-2 and PlGF and CD14 protein expression levels in the tumor with tumor size in 17 patients. The significance of their linear correlation is shown by Pearson r and p values. (**B-F**) Survival analysis. The good prognostic effect of the lower expression of CD14, ANG-2 and PIGF (B) is reflected by the clear separation from the over expression group on the Kaplan-Meier plot, with a P value of 0.0257 from a Log-rank test (F), whereas only ANG-2 (C) or PIGF (D) combined with CD14 separate worse lower and over expression groups, suggesting a synergistic effect of ANG-2 and PIGF to promote CD14-mediated angiogenesis and the corresponding impact on patient relapse free survival. (E) Shows the survival curve of lower expression patients for the three cases.

To this end we analyzed a dataset including tumor expression profiles and clinical data of 1809 breast cancer patients [50] and compared two subsets of patients: those with lowest and highest expression values for ANG-2, PIGF and CD14 (as TEM marker), using as threshold the first and fourth quartile respectively. These quartiles were computed independently for each gene, and the two groups of selected patients resulted from the intersection of them all (Fig. 7B-F). The Kaplan-Meier plot showed a clear separation between patients with low (n = 40) and high (n = 62) expression for these three genes, with a p-value of 0.0257 derived from log-rank analysis (Fig. 7B and F). Interestingly, we observed that the same analysis repeated for patients with high and low levels of ANG-2 and CD14 or PIGF and CD14 (and not for the remaining gene) resulted on p-values not statistically significant (0.0587 and 0.521 respectively, Fig. 7C-E), suggesting that the synergistic effect of the corresponding pathways is required to have a significant impact on the survival.

These results suggest that TEM infiltrating a tumor microenvironment enriched in Ang-2 and PlGF, which synergistically trigger TEM angiogenic activity through Tie-2 and VEGFR-1 (Fig. 4 and 5), may contribute to a worse patient survival. Further, tumor size correlated positively with the amounts of PlGF and Ang-2 content in the tumor microenvironment (Fig. 7A, P<0.01) while no significant correlation was observed with VEGF, Ang-1, MCP-1, SDF-1, TGF-α and TNF-β. Moreover, we measured by reverse phase protein arrays that in tumors the extent of TEM infiltration was significantly and linearly correlated with PLGF content (Fig. 7A) thus highlighting that Tie-2 and VEGFR-1 axes, as well as their cognate angiogenic TEM ligands Ang-2 and PlGF represent attractive therapeutic targets in breast cancer.

## Discussion

The key relevance of this study is a comprehensive understanding of the behavior of TEM in breast tumor vascularization. This goal was achieved by constructing an integrative and predictive model of TEM behavior based on experimental data. This model was interrogated to identify combined treatments that would alter TEM pro-angiogenic activity. Quite remarkably, four of the five predicted combined treatments that we validated experimentally proved to be extremely efficient at inhibiting or promoting tumor TEM proangiogenic activity, demonstrating the robustness of our model. Furthermore, this study demonstrates that the synergistic effect of these treatments relies on crosstalk between TNF-R1, VEGFR-1, TGF-β and TIE-2 pathways resulting in altered angiogenic activity (Figs. 2, 4 and 5), modulated expression of angiogenic receptors (Fig. 4A) and shifted paracrine profile (Fig. 6C).

Taken together, our results highlight crosstalks between TIE-2, VEGFR-1, TGF-β and TNF-α pathways of outstanding importance to promote (TNF-α/ANG-2/PlGF) or abrogate (TGF-β/TIE-2 inhibitor/VGFR1 or TIE-2 ligand) patient TEM pro-angiogenic activity.

Another contribution of this study is an effective approach to model relatively sparse data from distinct individuals (newborns and patients), who are inherently heterogeneous in nature. This challenge was overcome by a sustained and tight collaboration between the experts in the fields of computational and experimental sciences throughout all steps of the workflow (Fig. 1C). By setting up a rigorous experimental design we identified coherent variations and links across biological replicates and data sets, which provided a robust basis to reconstruct the TEM signaling network (Fig. 3). Furthermore, the modeling framework was an integral part of our experimental strategy, enabling the model predictions to address the biological questions, an issue that is of particular importance in systems biology [1,51,52]. In a traditional approach, it would have been unfeasible to experimentally test the complete set of up to three simultaneous perturbations using 12 distinct ligands, which would have led to 596 ligand combinations. The physiologically relevant combinations of ligands were discovered by applying the recently proposed MIS algorithm [43,44] to predict all minimal perturbations in the inferred regulatory network that can transition TEM into desired steady states (Table 4). The *in silico* minimal perturbations predicted by applying the MIS algorithm on the inferred ivdTEM regulatory network comprised not only a handful of the set of perturbations (or ligand combinations) and they were all shown to be experimentally valid when tested on ivdTEM and patient TEM (Figs. 4 and 5). The *in silico* prediction algorithm helped us to focus on the most clinically relevant monocytic ligands and to unravel treatments abrogating TEM pro-angiogenic activity at breast tumor sites. These results highlight the importance of mutual relationship between experimental and computational sciences. Furthermore, the combined computational and experimental approach followed in this study may provide a general strategy to study the behavior of limited cell subsets from patients in cancer and other diseases.

The main outcome of this modeling strategy for experimental and clinical oncology is the validation of treatments abrogating tumor TEM pro-angiogenic activity and thus simultaneously revealing their functional plasticity. Our study shows that treatments targeting TEM plasticity may constitute a valid therapeutic strategy to shift TEM to acquire a more anti-tumor M1-like phenotype. Moreover, the relapse free survival analysis showed a clear separation between patients with low and high expression for pro-angiogenic genes (ANG-2 and PIGF), and suggested that the synergistic effect of the corresponding pathways is required to have a significant impact on the survival.

Overall, our results obtained by employing the combined modeling and experimental approach suggest novel treatments for abrogating tumor TEM pro-angiogenic activity and reveals the functional plasticity of TEM.

## Materials and Methods

A detailed procedure of the methods used is provided as supplementary information in S1 Text.

### Ethics statement

This study was approved by the ethics committee of the University Hospital of Lausanne (reference number 170/07). Patient or subject tissue specimens were obtained according to the declaration of Helsinki and upon written informed consent.

### Patient and tissue specimens

A series of 40 primary invasive breast carcinoma specimens (Table 1) were resected from patients with breast cancer and tumors enzymatically dissociated as described [53]. All patients underwent surgery and sentinel node biopsy before treatment. The presence of nodal metastases and tumor pathological features were confirmed histologically and are detailed in Table 1. All patients were untreated before surgery. Peripheral blood was collected before surgery and processed as described [54].

### TEM differentiation and treatment

CD34^+^ hematopoietic progenitors from cord blood were isolated by immunomagnetic selection (StemCell Technologies Inc.) and cultured as previously described [40]. At day 6, cells were activated for 2h at 37°C with 100 ng/ ml of recombinant ligands (TNF-α 20 ng/ ml), washed and cultured for 36h in RPMI containing 10% FCS. When two treatments were successively applied, TEM were first exposed for 2h to a combined treatment of PlGF/TNF-α/ANG-2, washed and kept in culture for 30h in RPMI containing 10% FCS. They were then exposed to inhibitory treatments for 2h, washed and kept 24 hours longer in culture. TEM phenotype, cytokine secretion and pro-angiogenic activity were assessed by flow cytometry and *in vivo* or *in vitro* vascularization assay, respectively. Alternatively TEM were isolated from patient peripheral blood or tumor by CD14 immunomagnetic selection, exposed to treatments for 36h (30h exposure to angiogenic and inflammatory factors followed by 6h exposure to TIE-2 kinase inhibitor at 8 μM) and extensively washed. TEM viability was not affected under the different conditions of stimulation used and was > 95%

### Experimental design

We undertook a rigorous experimental design consisting in profiling changes in phenotype, cytokine secretion, gene expression and angiogenic activity from the same cell sample in response to treatments. Changes induced by the treatments were normalized to untreated cell in each biological replicate and cumulated across the treatments. In this study, a biological replicate was a population of ivdTEM from a distinct cord blood and exposed to the same treatment. Hence, biological replicates originated from distinct individuals (newborn or patients). This experimental design was kept rigorously for each biological replicate to allow analysis across biological replicates and data sets.

### Analysis of cell phenotype and cytokine secretion by flow cytometry

Following blocking of Fc receptors with antibodies, cells were labeled with CD14 (PerCP-Cy5.5), CD11b (FITC), TIE-2 (Alexa 647), VEGFR-1 (PE), TGFBR-1 (Pacific Blue), TNF-R1 (Pacific Orange), CXCR4-, CCR5-, α5β1-biotinylated specific antibodies (followed by streptavidin-Marina Blue) and analysed by flow cytometry using a Facs LSRII (BD Biosciences) equipped with a 610/20 nm filter on the violet detector. Pacific Blue and Pacific Orange NHSE (Invitrogen) were used to couple TGFBR-1 and TNF-R1-specific antibodies respectively as well as the corresponding isotype controls. The cell populations were manually examined based on their CD14 and CD11b intensities to identify DN, SP and DP cell populations and the frequency count and a mean intensity value for each channel were calculated. Secreted cytokines and angiogenic factors were quantified in cell conditioned medium using FlowCytomix technology (Bender MedSystems and RnD). Importantly, VEGF and PlGF were used as treatments (see *in vitro* TEM differentiation and treatments above) and also measured in conditioned medium as angiogenic factors secreted by TEM in response to the treatments (this section).

### *In vivo* and *in vitro* angiogenesis assay

Mouse experiments were approved by the veterinary service of Vaud Canton. The bacterial lipopolysaccharide membrane receptor CD14 is a component of the innate immune system mainly expressed by monocytes and macrophages and commonly used as a marker of these cell populations. Monocytes were isolated by CD14 immunomagnetic selection from patient tissue. For *in vivo* corneal vascularization assay, 20,000 CD14^+^ cells isolated by positive immunomagnetic selection (Stemcell Technologies) from peripheral blood (purity>95%) or dissociated tumors (purity>85% with no detectable CD45^-^ contamination) were injected (5μl) into the stromal part of the corneas of anesthesized NOD-scid IL2Rγ^null^ mice [39] using a 35 gauge nanofil injection kit (WPI, Stevenage, UK). Cornea vascularization was monitored with a digital stereomicroscope (Leica). Mice were euthanized 25 days post-injection and isolated eyes were fixed in 4% PFA, cryoprotected in a 30% sucrose solution and embedded in Yazulla media (30% egg albumin, 3% gelatin). Vascularization was assessed by immunostaining of the sagittal sections (10μm) with CD31-specific antibodies (Platelet Endothelial Cell Adhesion Molecule-1, PECAM-1) using a Zeiss motorized Axio Imager M1 fluorescent microscope. Retina was used as a positive control in all CD31 stainings. Quantification was performed with Image J software by measuring the fraction of the iris and cornea surface area containing vessels (CD31 positive surface areas of the iris and cornea/ surface area of the iris and cornea). This ratio was set up at 1 for the control eyes (no cell injected) and the data were normalized to the control (AU). *In vitro* angiogenesis sprouting assay was performed with HUVEC spheroids as previously described [40]. The corneal angiogenesis assay is still considered one of the best *in vivo* assays [55]. However, the surgical procedure is technically difficult and the assay time consuming. Therefore, we use *in vitro* angiogenesis sprouting assay [56] to assess the impact of multiple treatments and we validated the most relevant one *in vivo*.

### Reagents and antibodies

Chemicals unless indicated otherwise were from Sigma-Aldrich. Common stocks of cytokines, inhibitors and assay reagents were used to minimize experimental variability. Human recombinant cytokines were purchased from PeproTech (London, UK) and R&D Systems. All the antibodies used are listed in S1 Table. TIE-2 kinase inhibitor compound 7 was from Alexis Biochemicals (San Diego, CA).

### Statistical analysis and data treatments

Statistical analysis was performed using GraphPad Prism version 4.00 for Windows, GraphPad Software, San Diego, California, USA. Unless indicated differently, T test was used to determine p values. A p value < 0.05 was considered statistically significant. All data shown are means ± standard deviation. The effect of each treatment on the phenotype and secretions of TEM was calculated as the log_2_ of the mean fluorescence intensity (MFI) percent change compared to untreated cells and a heatmap produced with R (http://www.R-project.org).

### Estimation of the relative contribution of each cell population in the total cytokine production

Cumulated secretions from DN, SP and DP (i.e. ivdTEM) were measured experimentally and the secretions of each population mathematically inferred. For a given treatment, let Na, Nb and Nc be the relative number of cells present in each population (a = DN, b = SP, c = DP) and K be the amount of cytokine experimentally measured and expressed as a percentage of change of cytokine secretion to untreated cells. Then the relative contribution C of each population was expressed as:

Ca×Na+Cb×Nb+Cc×Nc=K

Using three independent biological replicates (1, 2, 3) and their associated cytokine measurements (K1, K2, K3) we could write the following three equations:

Ca×Na1+Cb×Nb1+Cc×Nc1=K1

Ca×Na2+Cb×Nb2+Cc×Nc2=K2

Ca×Na3+Cb×Nb3+Cc×Nc3=K3

The unknowns were Ca, Cb, and Cc while Na, Nb and Nb have been obtained from cytofluorimetry data. These equations were solved for all possible combinations of 3 biological replicates (4 to 84) and the median of the obtained C coefficients calculated. The coefficients were then used to infer the amount of cytokine by DN, SP and DP cell populations. In the few instances, the inferred cytokine amount was lower than 0% of untreated cells and the predicted value was set to a minimal positive value (1%).

### Identification of the links between treatment, receptor and cytokine

Within the TEM regulatory network, a link represents an effect (increase or decrease) on either receptor expression or cytokine secretion in response to single or combined ligands (Fig. 3). Of note, some treatments and secreted cytokines are identical. We applied the three following criteria to identify a link between treatment/receptor/cytokine. We retained only the links that are reproducible, of sufficient amplitude and coherent. Only 8% of the possible links matched these three criteria and were retained to construct the TEM regulatory network. A link was considered as reproducible when the treatment induced a reproducible effect across at least 3/4 of the biological replicates. Second, a treatment was retained if it induced a change of sufficient amplitude, i.e. when the effect of the treatment on receptor and cytokine was among the upper (the treatment increases the expression of a receptor or a cytokine) or lower (the treatment decreases the expression of a cytokine or a receptor) quartile of variation. Third, only coherent links were retained meaning that they should be always correlated or anti-correlated across the treatments, and not sometimes correlated and other times anti-correlated. As an example, a receptor A and a cytokine B are linked when they show similar or/and opposite variations across the treatments e.g. receptor A up-regulated)/ cytokine B up, and/or receptor A down/cytokine B down. The list of the retained links is provided in S4 Table.

### Computing minimal all perturbations sets in ivdTEM regulatory network

The interactions between treatment/receptor/cytokine as predicted by our experimental and computational approach (see previous section) were used to generate the dynamical regulatory network of ivdTEM (Boolean equations are provided in S6 Table). The MIS algorithm proposed in [43,44] was then applied to compute all possible minimal perturbation sets to force the network into desired steady state or phenotype. The MIS algorithm starts by unrolling the inferred regulatory network of ivdTEM into a tree-like structure starting from the nodes which have a fixed polarity (i.e. either high or low) in the desired final steady state. In the angiogenesis model of ivdTEM, the nodes corresponding to TIE-2 and VEGFR-1 are assigned a fixed polarity of either both high or both low for highly pro-angiogenic and weakly pro-angiogenic steady states respectively. The nodes with the fixed polarity are referred to as the root nodes of the network. The network is unrolled along a path in the regulatory network until a duplicate node is found. At that instance the unrolling process is terminated along this path. Once the network is unrolled along all paths originating from the root node, the MIS patterns are generated by scanning this unrolled network in two iterations. In the first iteration, the required polarity (i.e. over-expression or knock-down) of each node is propagated from the root node to the leaf nodes. In the second iteration, possible perturbations that can lead to the required polarity at each node are listed by scanning the unrolled network in the reverse order from the leaf nodes towards the root node in the breadth first manner. When two paths merge at one node, then only those MIS vectors that are compatible (i.e. the same node is not over-expressed on one path and knocked-down on the other path) along both the paths are taken into consideration while scanning the rest of the network. This process when terminates at the root node, only MIS vectors that have been compatible throughout the unrolled network shall remain in the list. The MIS algorithm does not involve explicit enumeration of perturbation patterns but rather generates these patterns by traversing the topology of the network ensuring that only the patterns leading to a desired cellular behavior are generated. Detailed methodology for generation of the MIS vectors and the network unrolling process are further described in [43,44].

### Gene expression profiling

Total RNAs from 100 000 monocytes were isolated and purified with the Qiagen RNeasy micro plus kit. RNA samples were hybridized to Affymetrix Human Gene 1.0 ST Arrays and images were processed to obtain probe intensities using standard procedures at the GTF (Gene Technology Facility, CIG, University of Lausanne). Background subtraction, RNA normalization and probeset summarization were performed using the Affymetrix Power Tools software package (Affymetrix CEL files). Sample correlation was performed on the top 1000 expressed probesets using Bioconductor *affy* and *affyPLM* packages in R [57]. This analysis indicated separate clustering of VEGF/TNF-α and ANG-2 or PIGF/TGF-β samples. Differentially expressed genes between different treatments were detected by fitting linear models and computing empirical Bayes moderated t statistics, comparing two groups at a time, using the *limma* package in R [58]. P values were adjusted for multiple comparisons using the Benjamini Hochberg procedure [59] and genes with an adjusted p value of < = 0.05 were selected as differentially expressed. For pathway analysis, differentially expressed genes were ranked according to fold change (high to low) comparing two treatments and Gene Set Enrichment Analysis (GSEA) was performed on the ranked lists against MSigDB gene sets using the NCBI gene id as a unique identifier [60]. Enrichment p values were adjusted for multiple comparisons using the Benjamini Hochberg procedure [59]. The microarray data from this publication have been submitted to the GEO database http://www.ncbi.nlm.nih.gov/geo/info/linking.html and assigned the identifier GSE34559.

### Survival analysis

Publicly available normalized expression data from 1809 breast cancer patients was downloaded from http://kmplot.com [50]. For the relapse free survival analysis we selected lowest and highest expression values of 205572_at, 209652_s_at and 201743_at probes, corresponding with ANG-2, PIGF and CD14 genes respectively, using as threshold the first and third quartile respectively. These quartiles were computed independently for each gene, and the two groups of selected patients resulted from the intersection of them all (see Fig. 7). To generate the Kaplan-Meier plots and to evaluate the separation between groups (log-rank statistic) we used the *survival* package in R. Tables with the resulting data and R scripts are included in supplementary table 7.

## Supporting Information

S1 Text Tumor tissue processing, TEM differentiation *in vitro*, TEM stimulation, *in vitro* angiogenesis assay, migration assay, tumor growth inhibition assay and protein profiling in tumor tissues. (DOCX)

S1 Fig TEM co-express Tie-2 and VEGFR-1. (**A**) Gating strategy of monocytes in patient peripheral blood and dissociated breast tumor. CD11b^+^, CD14^+^ cells are gated from live and single cell population and the expression of Tie-2 and VEGFR-1 was assessed in this population either in peripheral blood (A) or dissociated tumors (**B**). Isotype control antibodies were used to assess the expression of Tie-2 and VEGFR-1 in peripheral blood (A) and dissociated tumors (**B**). (**C**) In breast tumor tissue more than 95% of TEM co-express Tie-2 and VEGFR-1 as shown by confocal microscopy images of sections of frozen breast carcinomas and Facs analyses (B).
(TIFF)

S2 Fig *In vitro* differentiated TEM: characterization at resting state and upon stimulation. Patient TEM are (CD11b^-^, CD14^+^ i.e. double positive DP cells) while *in vitro* differentiated cells encompassed three cell populations: DN: double negative (CD11b^-^, CD14^-^), SP: single positive (CD11b^-^, CD14^+^) and DP: double positive (CD11b^+^, CD14^+^). The frequency of these population are as follows: DN (53.7%±10.8), SP (30.4%±11.9), DP (16.1%±5.6). *In vitro* differentiated TEM correspond to the DP cell population and display a phenotype and functions intermediate to blood and tumor patient TEM (Fig. 1A and B and Tables 2 and 3). (**A**) The expression of receptors at the surface of TEM differentiated *in vitro* was measured by flow cytometry at resting state in DN, SP, and DP (i.e. TEM) cell populations. Shown are cumulated data of 10 independent experiments. Box plots represent values between 25^th^ and 75^th^ percentile with a line at the median (50^th^ percentile). The whiskers extend above and below the box to show the highest and the lowest values. Significant variations between SP and DP cell phenotypes are indicated with asterisks in the DP box plots (* P < 0.05, ** P < 0.01, T Test). (**B**) *In vitro* differentiated cells were exposed to different combinations of ligands and changes in receptor expression at the surface of DN, SP and DP (i.e. TEM) cell populations were measured by flow cytometry 36 hours post-treatment and displayed as mean log_2_ ratios relative to untreated cells. Shown are cumulated data of 3 to 9 independent experiments. Significant variations (P < 0.05, T test) in VEGFR-1 and TIE-2 expression in SP and DP cell populations are indicated with an asterisk in the heatmap. The corresponding experimental data and all P values are available in S2 Table. (**C**) Secretion of cytokines and angiogenic factors in response to treatments in DN, SP and DP (i.e. TEM) cell populations differentiated *in vitro*. In contrast to receptor expression, TEM secretions were released in the culture medium and could not be measured in individual cell populations. Thus, cumulated TEM secretions from DN, SP and DP cell populations were measured experimentally and the secretions for each individual population were mathematically inferred (see Materials and Methods) and displayed as mean log_2_ ratios relative to untreated cells. Angiogenic factors are boxed. Shown are cumulated data of 5 to 10 independent experiments. The corresponding experimental data are available in S2 Table.
(TIFF)

S3 Fig Impact of PlGF/TGF-β/TIE-2inhibitor treatment on ivdTEM functions. ivdTEM were left untreated or treated with PlGF/TGF-β/TIE-2inhibitor and (**A**) their aptitude to migrate towards MDA-231 breast epithelial cells assessed *in vitro* (n = 3), (**B**) their ability to slow the growth of MDA-231-GFP cells was measured in a 48h co-culture assay (n = 3). (**C**) *In vitro* angiogenic assay: representative images of HUVEC cell sprouting in the presence or absence of ivdTEM.
(TIFF)

S1 Table Antibodies used for the study listed by application. (DOCX)

S2 Table Expression of receptors at the surface of TEM differentiated *in vitro* in response to treatments. Changes are expressed relative to untreated cells (untreated cells: 100%). Significance of these changes are indicated in a P value table (T test) for SP and DP cell populations.
(XLS)

S3 Table Changes in cytokine secretion experimentally measured in the conditioned medium of TEM differentiated *in vitro* in response to treatments (untreated cells: 100%). (XLS)

S4 Table Links between treatment, receptor and cytokine in DP cell population i.e. TEM. All the links retained for the construction of TEM dynamical regulatory network are shown.
(XLS)

S5 Table List of genes from *in vitro* differentiated TEM displaying significant variations of their expression in response to ANG-2/TGF and PlGF/TGF treatments. Genes specifically regulated by each of these treatments were classified in PlGF/TGF and ANG-2/TGF unique lists. Genes affected by both treatments were listed in ANG-2/TGF PLGF/TGF intersect.
(XLSX)

S6 Table Boolean equations used for representing TEM regulatory network shown in Fig. 3. (TXT)

S7 Table R scripts and normalized expression and clinical data from 1809 breast cancer patients to perform the survival analysis and generate Kaplan-Meier plots. (XLS)
