# Supplementary material for: Angiogenic Activity of Breast Cancer Patients’ Monocytes Reverted by Combined Use of Systems Modeling and Experimental Approaches
Source: PLoS Comput Biol. 2015 Mar 13;11(3):e1004050. doi: 10.1371/journal.pcbi.1004050 (PMC4359163; doi:10.1371/journal.pcbi.1004050)
Supplement: S1 Text — (DOCX) [file pcbi.1004050.s001.docx]

**Supplementary material and methods**

**Tumor tissue processing**

The tumor tissues were processes under sterile conditions. Fresh sterile tumor tissue were chopped with a scalpel into 2 mm fragments and transferred into a 50 ml Erlenmeyer containing RPMI supplemented with 0.1% collagenase I (Worthington, NJ; 10 ml solution for 1g of tissue). The mixture was stirred gently for 45 min at 37°C in a tissue culture incubator. The collagenase activity was stopped by adding FCS at a final concentration of 10%. The mixture was filtered through a 70 µm mesh, washed by centrifugation using RPMI containing 10% FCS and used for immunomagnetic selection.

**TEM differentiation *in vitro***

CD34 progenitors cells were isolated using EasySep selection kit (StemCell Technologies Inc.) from umbilical cord blood obtained from newborn babies of healthy volunteer pregnant women according to the declaration of Helsinki. Purified CD34^+^ cord blood hematopoietic progenitor cells were cultured in a 24-well tissue plate in RPMI1640 medium containing 10% FCS, 10% conditioned medium from MCF-7 cell line, 50 ng/ml of Flt3 ligand, 50 ng/ml of SCF (Stem Cell Factor), 10 ng/ml of thrombopoietin (all these recombinant human proteins from AbD Serotec) and 10ng/ml of IL-6 (Peprotech). Cell cultures were harvested at day 6 or 7.

**TEM stimulation**

At day 6 or 7, the cell culture was harvested and resuspended in PRMI1640 medium containing 2% FCS at a concentration of 1 million/ml for 2h at 37°C. The cells were exposed to the different ligand/ TIE-2 kinase inhibitor (at a final concentration of 8 µM) combinations for 2h at 37°C. All recombinants ligands were used at 100 ng/ ml but TNF-α (20 ng/ ml). The cells were washed and cultured for 36h in RPMI containing 10% FCS. When two treatments were successively applied, TEM were first exposed for 2h to a combined treatment of PlGF/TNF-α/ANG-2, washed and kept in culture for 30h in RPMI containing 10% FCS. They were then exposed to inhibitory treatments for 2h, washed and kept 24 hours longer in culture. At the end of the stimulation, the cells were extensively washed and kept in RPMI1640 containing 10% FCS. TEM phenotype and cytokine profile were assessed by flow cytometry using of cells and conditioned medium, respectively.

***In vitro* angiogenesis assay, migration assay and tumor growth inhibition assay**

Monocytes were isolated from *in vitro* culture or patient specimen (peripheral blood or tumor) using CD11b or CD14 immunomagnetic selection, respectively. We used anti-human CD11b-PE or anti-human CD14-PE followed by a PE selection kit (StemCell Technologies Inc.). Immunomagnetically isolated cells were stimulated as described above, extensively washed and used for functional assays.

*In vitro* angiogenic assay:

This assay measures the aptitude of HUVEC to form tubes *in vitro* (sprouting assay) in response to angiogenic monocytes. This sprouting assay is done in a 3D culture and consists of HUVEC grown on microcarrier beads and embedded in a 3D fibrin gel, as previously described [[40](#_ENREF_40)]. HUVEC (passage 2 to 6) were grown in endothelial cell (EC) medium consisting of EBM-2 medium from Lonza with supplements. Cytodex microcarrier beads (Sigman C3275) are swollen in PBS for 3h at RT and sterilized by autoclaving for 15 min at 115°C. About 400 HUVEC/ bead are required and 12-15 beads are used per well of a 96 well plate. The number of beads needed are washed three times by gravity in RPMI1640 supplemented with 10% FCS using a 14 ml Falcon tube (352059) and resuspended in 0.5 ml of RPMI supplemented with 10%. FCS. HUVEC were trypsinized and the required number of cells resuspended in 0.5 ml of EC medium and added to the beads. Beads and HUVEC were incubated for 5h at 37°C and gently shake manually every 20 min and then supplemented with 1 ml of RPMI10% and 1 ml of EC medium and incubated overnight at 37°C. The next day, the beads are homogeneously covered with HUVEC, the quality of the beading is assessed by observation with a microscope. HUVEC coated beads are washed twice with EC medium (pre-warmed at 37°C) and resuspended in a solution of 2 mg/ml of fibrinogen containing 50ng/ml of Aprotinin (Sigma A3428) at a final concentration of 275 beads/ml. 40 µl of HUVEC-coated beads are distributed in a well of a 96 well plate containing 0.8µl of Thrombin (Sigma T9549) using a cut pipet tip. The fibrin clots within the next 15 min at 37°C. During this time, normal human fibroblasts (NDHF) are trypsinized and added to the top of the fibrin gel, 300 fibroblasts (in 15µl) are needed per well of a 96 well plate and are used as feeder cells. Finally, monocytes -previously isolated and stimulated as described above- were extensively washed and added to the top of the fibrin gel (10 000 monocytes/well of a 96 well plate, in a maximum volume of 15µl). The wells are fed with EC medium every other day and HUVEC sprouts are visible after 2 days of culture. Each well contains 12-15 beads that are imaged at day 2 and 5. Representative images are shown in Supplementary Figure 2C. Average cumulated sprout length is used for quantification. It represents the mean of the sprout length measured on 10 distinct beads/condition of stimulation. Shown data are corrected for background sprouting which represent the basal sprouting of HUVEC in the absence of monocyte (<10 % of maximal sprouting intensity obtained in the presence of angiogenic monocytes).

Migration assay: MDA-231 cells (breast epithelial cell line) were plated in a 96 cell plate and used when confluent. The medium was removed and a layer of 0.1 ml of 0.6 % methylcellulose was placed on top of the cells. 90 min later, 5000 CD11b^+^ immunomagnetically selected monocytes (stimulated or left unstimulated) were added on the top. In order to visualize easily the tumor cells and the monocytes, we used MDA-231-GFP and monocytes were stained with the cell tracker PKH-26 (red fluorescent dye) according to the instructions of the supplier. Tumor cell factors form a gradient into the layer of methycellulose and attract the monocytes to the tumor. The number of monocytes reaching the tumor cell layer was quantified by imaging the layer of tumor cells with an epifluorescent microscope at different time point over the first 4 hours of migration.

Growth inhibition assay. MDA-231 cells were plated in a 96 cell plate at 20% confluence in the presence of 25 000 CD11b^+^ immunomagnetically selected monocytes treated or untreated. MDA-231-GFP cells were used and the growth of tumor cells over 48 hours was evaluated by quantifying the surface covered by tumor cells using an epifluorescent microscope.

**Protein profiling in tumor tissues**

Snap-frozen fragments of tumor tissues were crushed in liquid nitrogen using a mortar and a pestle The proteins from the resulting tissue powder sere solubilized using Tissue Protein Extraction Reagent (Thermo Fisher, Waltham, MA)/ SDS buffer (125 mM Tris, pH 6.8 containing 4% SDS, 10% glycerol and 2% 2-mercaptoethanol), boiled and printed onto nitrocellulose slides for reverse phase protein (RPP) arrays. The printing and the detection were performed as previously described ([Brembilla NC *et al*, Proteomics 2009](#_ENREF_8); [Brembilla NC *et al*, Blood 2008](#_ENREF_8)) using specific antibodies for CD14 (Abnova), PlGF and Ang-2 (Genetex).
