# Supplementary figures and images for: Angiogenic Activity of Breast Cancer Patients’ Monocytes Reverted by Combined Use of Systems Modeling and Experimental Approaches
Source: PLoS Comput Biol. 2015 Mar 13;11(3):e1004050. doi: 10.1371/journal.pcbi.1004050 (PMC4359163; doi:10.1371/journal.pcbi.1004050)

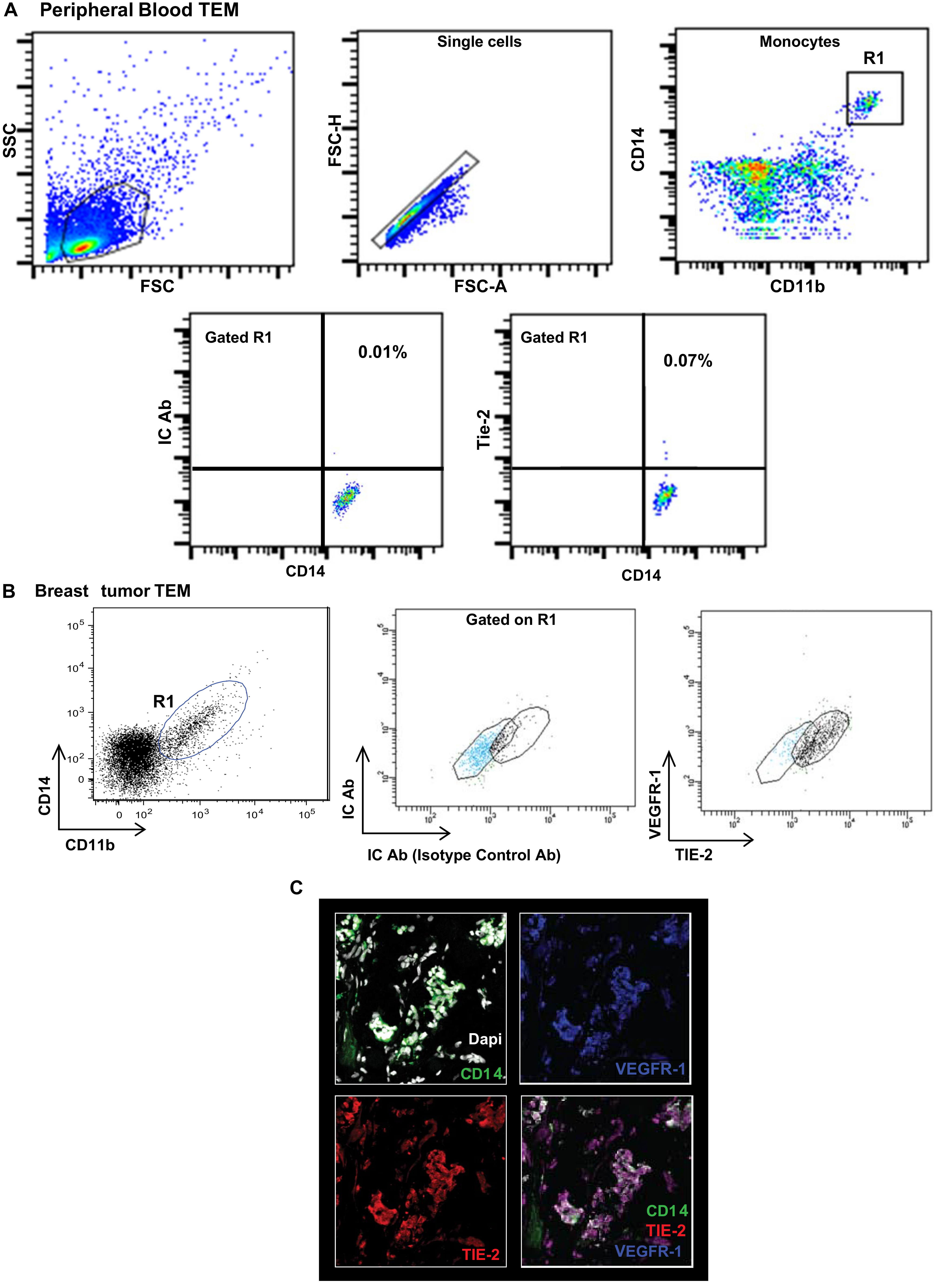

Supplement: S1 Fig — (A) Gating strategy of monocytes in patient peripheral blood and dissociated breast tumor. CD11b+, CD14+ cells are gated from live and single cell population and the expression of Tie-2 and VEGFR-1 was assessed in this population either in peripheral blood (A) or dissociated tumors (B). Isotype control antibodies were used to assess the expression of Tie-2 and VEGFR-1 in peripheral blood (A) and dissociated tumors (B). (C) In breast tumor tissue more than 95% of TEM co-express Tie-2 and VEGFR-1 as shown by confocal microscopy images of sections of frozen breast carcinomas and Facs analyses (B). (TIFF) [file pcbi.1004050.s002.tiff]

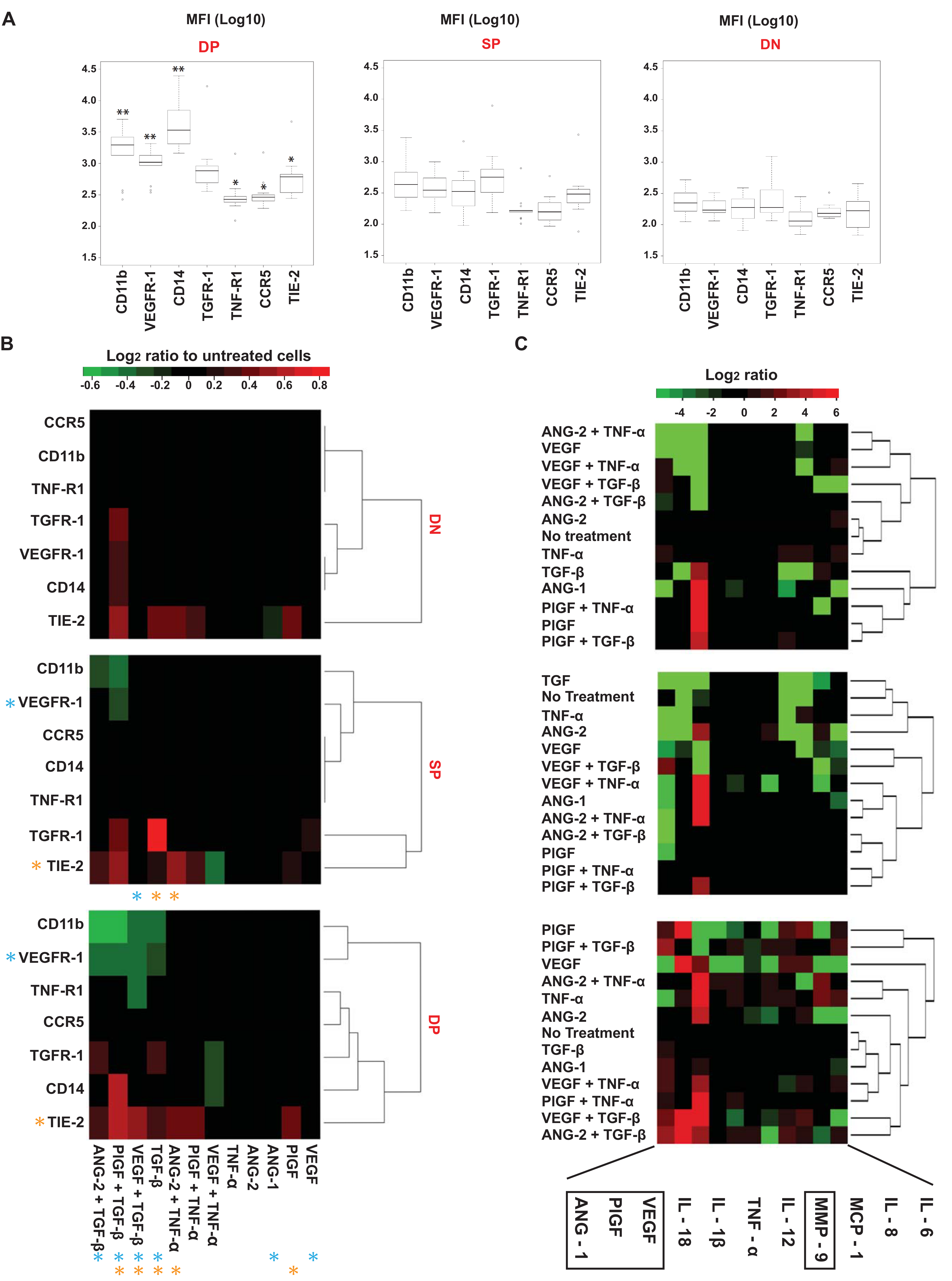

Supplement: S2 Fig — Patient TEM are (CD11b-, CD14+ i.e. double positive DP cells) while in vitro differentiated cells encompassed three cell populations: DN: double negative (CD11b-, CD14-), SP: single positive (CD11b-, CD14+) and DP: double positive (CD11b+, CD14+). The frequency of these population are as follows: DN (53.7%±10.8), SP (30.4%±11.9), DP (16.1%±5.6). In vitro differentiated TEM correspond to the DP cell population and display a phenotype and functions intermediate to blood and tumor patient TEM (Fig. 1A and B and Tables 2 and 3). (A) The expression of receptors at the surface of TEM differentiated in vitro was measured by flow cytometry at resting state in DN, SP, and DP (i.e. TEM) cell populations. Shown are cumulated data of 10 independent experiments. Box plots represent values between 25th and 75th percentile with a line at the median (50th percentile). The whiskers extend above and below the box to show the highest and the lowest values. Significant variations between SP and DP cell phenotypes are indicated with asterisks in the DP box plots (* P < 0.05, ** P < 0.01, T Test). (B) In vitro differentiated cells were exposed to different combinations of ligands and changes in receptor expression at the surface of DN, SP and DP (i.e. TEM) cell populations were measured by flow cytometry 36 hours post-treatment and displayed as mean log2 ratios relative to untreated cells. Shown are cumulated data of 3 to 9 independent experiments. Significant variations (P < 0.05, T test) in VEGFR-1 and TIE-2 expression in SP and DP cell populations are indicated with an asterisk in the heatmap. The corresponding experimental data and all P values are available in S2 Table. (C) Secretion of cytokines and angiogenic factors in response to treatments in DN, SP and DP (i.e. TEM) cell populations differentiated in vitro. In contrast to receptor expression, TEM secretions were released in the culture medium and could not be measured in individual cell populations. Thus, cumulated TEM secreti [file pcbi.1004050.s003.tiff]

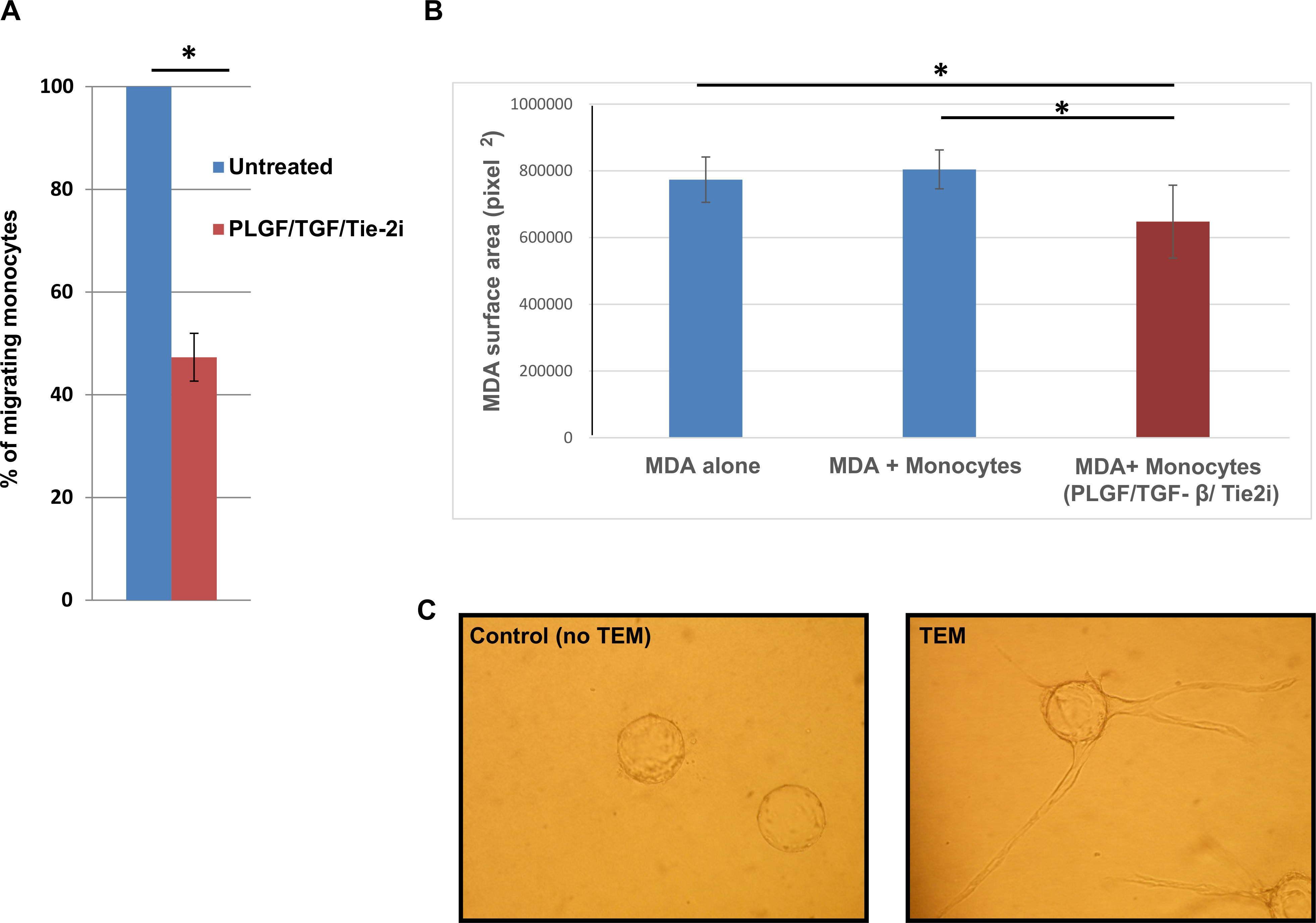

Supplement: S3 Fig — ivdTEM were left untreated or treated with PlGF/TGF-β/TIE-2inhibitor and (A) their aptitude to migrate towards MDA-231 breast epithelial cells assessed in vitro (n = 3), (B) their ability to slow the growth of MDA-231-GFP cells was measured in a 48h co-culture assay (n = 3). (C) In vitro angiogenic assay: representative images of HUVEC cell sprouting in the presence or absence of ivdTEM. (TIFF) [file pcbi.1004050.s004.tiff]
