# Supplementary material for: Angiogenic Activity of Breast Cancer Patients’ Monocytes Reverted by Combined Use of Systems Modeling and Experimental Approaches
Source: PLoS Comput Biol. 2015 Mar 13;11(3):e1004050. doi: 10.1371/journal.pcbi.1004050 (PMC4359163; doi:10.1371/journal.pcbi.1004050)
Supplement: S1 Table — (DOCX) [file pcbi.1004050.s005.docx]

|  | | **Antibodies description** | **Supplier** | **Product Reference** |
| --- | --- | --- | --- | --- |
| **Immunohistochemistry antibodies** |  | |  |  |
|  | Mouse anti-human CD31 | | Abcam | #ab9498 |
|  | Alexa 594-conjugated donkey anti-rabbit | | Invitrogen | #A-21207 |
|  | Alexa 647-conjugated donkey anti-goat | | Invitrogen | #A-21447 |
|  | Alexa 647-conjugated goat anti-mouse IgG1 | | Invitrogen | #A-21240 |
|  | Rat anti-mouse CD31 | | BD Pharmingen | #550274 |
|  | Alexa 568-conjugated goat anti-rat IgG | | Molecular Probes | #A-11077 |
|  | Goat anti-human TIE-2 | | R&D Systems | #AF313 |
| **Flow Cytometry antibodies** |  | |  |  |
|  | CD11b-FITC | | Biolegend | #301404 |
|  | CD14-PerCP-Cy5.5 | | Biolegend | #325622 |
|  | Tie-2-Alexa 647 | | Biolegend | #334210 |
|  | VEGFR1-PE | | R&D Systems | #FAB321P |
|  | CCR5-biotin | | R&D Systems | #FAB183B |
|  | CD49e-biotin | | Biolegend | #328005 |
|  | CXCR4-biotin | | Biolegend | #306504 |
|  | CD105-Pacific Blue | | Biolegend | #323202 |
|  | CD120a-Pacific orange | | BD Pharmingen | #550514 |
|  | Alexa647-mouse IgG1 | | Biolegend | #400130 |
|  | PE-mouse IgG1 | | Biolegend | #400112 |
|  | Biotin-mouse IgG2b | | Biolegend | #400303 |
|  | Biotin-mouse IgG2a | | Biolegend | # 400203 |
|  | Pacific Blue-mouse IgG1 | | BD Pharmingen | #610736 |
|  | Pacific Orange-mouse IgG2a | | Abnova | #H00004092-M07 |
|  | Streptavin Marina Blue | | Invitrogen | #S11-221 |
|  | Anti-human CD32 | | BD Pharmingen | #557333 |
|  | Anti-human CD32 | | Stemcell | #18520 |
|  | Goat anti- human Tie-2 | | R&D Systems | #AF313 |
|  | Rabbit anti- human VEGFR1 | | Abcam | #ab2350 |
|  | Donkey anti-rabbit Dylight 488 | | Jackson Immunoresearch | #711-486-152 |
|  | Alexa 647-conjugated donkey anti-goat | | Invitrogen | #A-21447 |

**Supplementary Table1:** Antibodies used for the study listed by application.
